# The genetic landscape of RAG deficiency: Novel and founder variants in a large Algerian cohort

**DOI:** 10.70962/jhi.20260168

**Published:** 2026-10-26

**Authors:** Brahim Belaid, Koon-Wing Chan, Jennifer Stoddard, Julie Niemela, Heather Kenney, Adriana A. De Jesus, Daniel Leung, Jing Yang, Xing Tian Yang, Abdelhak Dehimi, Ouardia Drali, Aida Mohand Oussaid, Lydia Lamara Mahammed, Lilya Meriem Berkani, Hassiba Sakhri, Asmaa Abed, Meriem Tahir, Fatma Merah, Alaa Hamidou Benmoussa, Ines Allam, Lylia Djidjeli, Hadia Boussaha, Amina Salmi, Sonia Yasmine Kirane, Radia Rezzak, Fella Lamrani, Souhila Melzi, Fella Dahmoune, Fairouz Guerrouche, Ahlem Mechali, Amina Saad Djaballah, Lamia Amirat, Meriem Oussalah, Yacine Inouri, Saadeddine Dib, Fatima Benbida, Amara Zelaci, Mohamed Amine Ifri, Kawthar Allali, Seddam Hocin Gafazi, Sara Makhloufi, Houda Larkat, Fadela Bendaoud, Noui Djamila, Hamza Boucenna, Khadidja Benallal, Chahrazed El Mezouar, Samia Bicha, Rachid Bouhdjila, Nadia Bensaadi, Farih Mehdid, Malek Benakli, Rawda Aboura, Zoulikha Zeroual, Leila Smati, Salih Bendeddouche, Reda Belbouab, Azzeddine Mekki, Belkacem Bioud, Samir Mohamed Ladj, Asmahane Ladjouze, Ourida Gacem, Nafissa Benhalla, Jaime Sou Rosa Duque, Sergio Rosenzweig, Yu Lung Lau, Luigi Daniele Notarangelo, Reda Djidjik

**Affiliations:** 1 Department of Immunology, Beni Messous University Hospital Center, Algiers, Algeria; 2 Faculty of Pharmacy, University of Health Sciences, Algiers, Algeria; 3 Department of Paediatrics and Adolescent Medicine, School of Clinical Medicine, Li Ka Shing Faculty of Medicine, The University of Hong Kong, Hong Kong, China; 4 Immunology Service, Department of Laboratory Medicine, Clinical Center, National Institutes of Health, Bethesda, MD, USA; 5 National Institute of Allergy and Infectious Diseases, National Institutes of Health, Bethesda, MD, USA; 6 Department of Pediatrics, Setif University Hospital Center, Faculty of Medicine, The University of Setif 1, Setif, Algeria; 7 Department of Pediatric Pulmonology and Allergology, Canastel Children’s Hospital, Faculty of Medicine, The University of Oran, Oran, Algeria; 8 Department of Pediatrics A, Beni Messous University Hospital Center, Algiers, Algeria; 9 Faculty of Medicine, University of Health Sciences, Algiers, Algeria; 10 Central Laboratory, Zeralda Burn Hospital, Algiers, Algeria; 11 Department of Pediatrics B, Beni Messous University Hospital Center, Algiers, Algeria; 12 Department of Pediatrics, Bab El Oued University Hospital Center, Algiers, Algeria; 13 Department of Pediatrics, Bejaia University Hospital Center, Faculty of Medicine, The University of Bejaia, Bejaia, Algeria; 14 Department of Pediatrics B, Hussein Dey University Hospital Center, Algiers, Algeria; 15 Department of Pediatrics, Bologhine Hospital, Algiers, Algeria; 16 Department of Pediatrics, Birtraria Hospital, Algiers, Algeria; 17 Department of Pediatrics, Beni Messous Military Mother and Child Hospital, Algiers, Algeria; 18 Department of Pediatrics, Specialized Mother and Child Hospital of Tlemcen, Tlemcen, Algeria; 19 Faculty of Medicine, University of Tlemcen, Tlemcen, Algeria; 20 Department of Pediatrics, El Oued Hospital, El Oued, Algeria; 21 Department of Pediatrics, Thenia Hospital, Boumerdes, Algeria; 22 Department of Pediatrics, Ouargla Hospital, Ouargla, Algeria; 23 Department of Pediatrics, Djelfa Hospital, Djelfa, Algeria; 24 Department of Pediatrics, Merouana Hospital, Batna, Algeria; 25 Department of Pediatrics, Boussaada Hospital, Boussaada, Algeria; 26 Department of Neonatology, Mother and Child Specialized Hospital of Batna, Algeria; 27 Faculty of Medicine, The University of Batna, Algeria; 28 Department of Pediatrics, Batna University Hospital Center, Batna, Algeria; 29 Department of Pediatrics, Sidi Bel Abbes University Hospital Center, Faculty of Medicine, The University of Sidi Bel Abbes, Sidi Bel Abbes, Algeria; 30 Department of Pediatrics B, University Hospital of Constantine, Faculty of Medicine, The University of Constantine 3, Constantine, Algeria; 31 Department of Pediatrics, Tizi Ouzou University Hospital Center, Faculty of Medicine, the University of Tizi Ouzou, Tizi Ouzou, Algeria; 32 Department of Hematology and Bone Morrow Transplantation, Pierre and Marie Curie Center, Algiers, Algeria; 33 Department of Pediatrics A, Hussein Dey University Hospital Center, Algiers, Algeria; 34 Department of Pediatrics, Tlemcen University Hospital Center, Tlemcen, Algeria; 35 Department of Pediatrics, Mustapha Bacha University Hospital Center, Algiers, Algeria

## Abstract

RAG1/RAG2 deficiency causes diverse SCID phenotypes, but large, systematically characterized cohorts from North Africa remain lacking. We retrospectively characterized 128 confirmed *RAG1*/*RAG2*-deficient patients diagnosed over 14 years in Algeria, classified per PIDTC 2022 criteria. Consanguinity was high (70.3%), with RAG2 (57.0%) outnumbering RAG1 (43.0%), a reversal of patterns reported elsewhere. Classical SCID (61.0%) and Omenn syndrome (23.4%) predominated. Two candidate founder variants (RAG1 c.519del, RAG2 c.104G>T) accounted for 76.6% of genotypes, yet identical c.519del genotypes produced three discordant phenotypes. Four novel variants and four digenic/oligogenic cases broadened the molecular spectrum. Despite a short diagnostic delay, mortality was 89.1%, driven primarily by near-absent HSCT access (2.3%). This study expands the molecular and clinical spectrum of RAG deficiency in an underrepresented population, revealing a reversed RAG1/RAG2 ratio and phenotypic heterogeneity despite shared founder genotypes. These findings prioritize functional validation of uncertain-significance variants and underscore the need for NBS and expanded transplant access to translate diagnosis into survival.

## Teaser

This study characterizes RAG1/RAG2 deficiency, a severe inherited immune disorder, in the largest reported cohort from a North African country. Two founder mutations explain most cases, yet limited transplant access drives high mortality, highlighting the need for newborn screening.

## Introduction

Severe combined immunodeficiency (SCID) comprises a heterogeneous group of life-threatening inborn errors of immunity (IEIs) characterized by a profound block in T lymphocyte development, uniformly fatal in infancy unless diagnosed and treated before overwhelming infection (1, 2). Among the recognized genetic causes of SCID, biallelic mutations in RAG1 and RAG2 underlie the T^−^B^−^NK^+^ subgroup, an estimated 20–30% of all SCID cases (3). RAG1 and RAG2 encode a heterodimeric enzyme that initiates V(D)J recombination, the somatic rearrangement assembling T and B cell receptor genes from variable, diversity, and joining segments; RAG1 provides the catalytic RNase H-like domain responsible for DNA cleavage, while RAG2 acts as an essential regulatory cofactor (4). Loss of this recombinase activity precludes a diverse antigen receptor repertoire, resulting in the profound or complete absence of mature T and, in most cases, B lymphocytes that defines RAG deficiency.

The clinical and immunological consequences of RAG1/RAG2 mutations span a broad spectrum, tracking the residual V(D)J recombinase activity retained by the mutant protein (4). Null or near-null mutations abolish activity entirely, producing classical SCID (c-SCID), a profound block in T and B cell development (T^−^B^−^NK^+^ SCID). Hypomorphic mutations, with environmental factors, can produce a broader range of phenotypes: Omenn syndrome (OS), with autologous, oligoclonal, activated T cells infiltrating skin and organs and autoimmune cytopenia; leaky SCID (l-SCID); and later-onset combined immunodeficiency (CID) with granulomas and autoimmunity, reflecting a continuum rather than a binary severe/mild distinction. The Primary Immunodeficiency Treatment Consortium (PIDTC) published revised consensus definitions in 2022, updating its 2014 criteria with contemporary genetic and immunological thresholds, e.g., lowering the CD3^+^ threshold for typical SCID to <0.05 × 10^9^/L and excluding maternal T cell engraftment to distinguish OS from l-SCID (5, 6).

Despite this general relationship, genotype alone does not fully determine clinical presentation. Identical RAG1/RAG2 mutations, including within the same sibship, can produce discordant phenotypes ranging from c-SCID to OS (7), and environmental or infectious exposures may further modulate immune dysregulation on an identical genetic background (8), indicating that additional, incompletely understood factors shape clinical expression. This has direct clinical consequences: hematopoietic stem cell transplantation (HSCT) remains the only curative treatment for RAG deficiency, and survival strongly depends on transplantation timing relative to infectious complications (9), making early, accurate diagnosis critical regardless of genotype.

Most large-scale characterizations of RAG1/RAG2 deficiency to date originate from North American, European, or other single-country referral centers, typically <40 patients (10, 11, 12, 13), except for a recent multi-center series pooling 157 patients across continents using multiomics to reveal distinctive immune dysregulation among the four subtypes (14). Middle East and North Africa (MENA) populations, where consanguineous marriage affects an estimated 20–50% of unions and recurrent founder mutations have been documented for several other autosomal recessive CIDs, remain underrepresented in the RAG deficiency literature (15). To our knowledge, no large-scale, dedicated clinical, immunological, and molecular characterization of RAG1/RAG2 deficiency has previously been reported from Algeria, despite its established tradition of consanguinity-associated founder mutations in other PIDs.

Here, we report the clinical, immunological, and molecular characterization of a cohort of 128 patients with genetically confirmed *RAG1* or *RAG2* deficiency, diagnosed over a 14-year period at Algeria’s principal national reference center for IEIs. This study aimed to characterize the clinical, immunological, and molecular spectrum of RAG deficiency in this population and to establish genotype-phenotype relationships and assess survival and access to curative treatment.

## Results

### General characteristics of the cohort

The cohort included 128 patients with genetically confirmed RAG1 or RAG2 deficiency, with a slight male predominance (70, 54.7%; P = 0.364). RAG2 variants were more frequent than RAG1 (73, 57% vs. 55, 43%). Parental consanguinity was reported in 90/128 (70.3%), most commonly second-degree (70/90, 77.8%), and family history of affected/deceased siblings in 85/128 (66.4%); neither differed significantly across phenotypes (P = 0.150, P = 0.115) (Table 1).

**Table 1.** General characteristics of the cohort

Variable	Overall (*n* = 128)	c-SCID (*n* = 78)	OS (*n* = 30)	l-SCID (*n* = 9)	CID (*n* = 11)	P value
**Gender**	​	​	​	​	​	0.364
M	70 (**55.7%**)	44 (56.4%)	14 (46.7%)	7 (77.8%)	5 (45.5%)	​
F	58 (45.3%)	34 (43.6%)	16 (53.3%)	2 (22.2%)	6 (54.5%)	​
**Causal gene**	​	​	​	​	​	<0.001
RAG2	73 (57.0%)	68 (87.2%)	0 (0.0%)	1 (11.1%)	4 (36.4%)	​
RAG1	55 (42.9%)	10 (12.8%)	30 (100.0%)	8 (88.9%)	7 (63.6%)	​
**Diagnostic timeline**						
Age at symptom onset, months	3.0 [1.6–4.0] (*n* = 110)	3.0 [2.0–4.0] (*n* = 70)	1.2 [0.2–2.9] (*n* = 22)	5.0 [2.0–5.5] (*n* = 7)	24.0 [11.5–54.0] (*n* = 11)	<0.001
Age at diagnosis, months	5.0 [3.0–7.0] (*n* = 122)	5.0 [4.0–6.0] (*n* = 76)	3.0 [2.1–5.0] (*n* = 26)	3.0 [3.0–9.0] (*n* = 9)	36.0 [23.0–78.0] (*n* = 11)	<0.001
Diagnostic delay, months	2.0 [1.0–4.0] (*n* = 109)	2.0 [1.0–3.4] (*n* = 70)	2.0 [1.5–2.5] (*n* = 21)	2.0 [0.8–4.0] (*n* = 7)	17.0 [12.0–30.0] (*n* = 11)	<0.001
**Consanguinity**	​	​	​	​	​	0.150
Yes	90 (70.3%)	57 (73.1%)	20 (66.7%)	8 (88.9%)	5 (45.5%)	​
No	38 (29.9%)	21 (26.9%)	10 (33.3%)	1 (11.1%)	6 (54.5%)	​
**Degree of consanguinity (among consanguineous, *n* = 90)**						
Second degree	70 (77.8%)	43 (75.4%)	15 (75.0%)	7 (87.5%)	5 (100.0%)	​
Thirrd degree	14 (15.6%)	10 (17.5%)	3 (15.0%)	1 (12.5%)	0 (0.0%)	​
Fourth degree	4 (4.4%)	3 (5.3%)	1 (5.0%)	0 (0.0%)	0 (0.0%)	​
Undetermined	2 (2.2%)	1 (1.8%)	1 (5.0%)	0 (0.0%)	0 (0.0%)	​
**Family history of affected/deceased siblings**	​	​	​	​	​	0.115
Yes	85 (66.4%)	52 (66.7%)	23 (76.7%)	3 (33.3%)	7 (63.6%)	​
No	43 (33.6%)	26 (33.3%)	7 (23.3%)	6 (66.7%)	4 (36.4%)	​

Data are presented as *n* (%) for categorical variables and as median [IQR] for continuous variables. P values compare RAG1 vs. RAG2 groups using the Mann–Whitney U test for continuous variables and Fisher’s exact test (2 × 2 tables) or Pearson’s chi-squared test for categorical variables.

**Figure S1.**
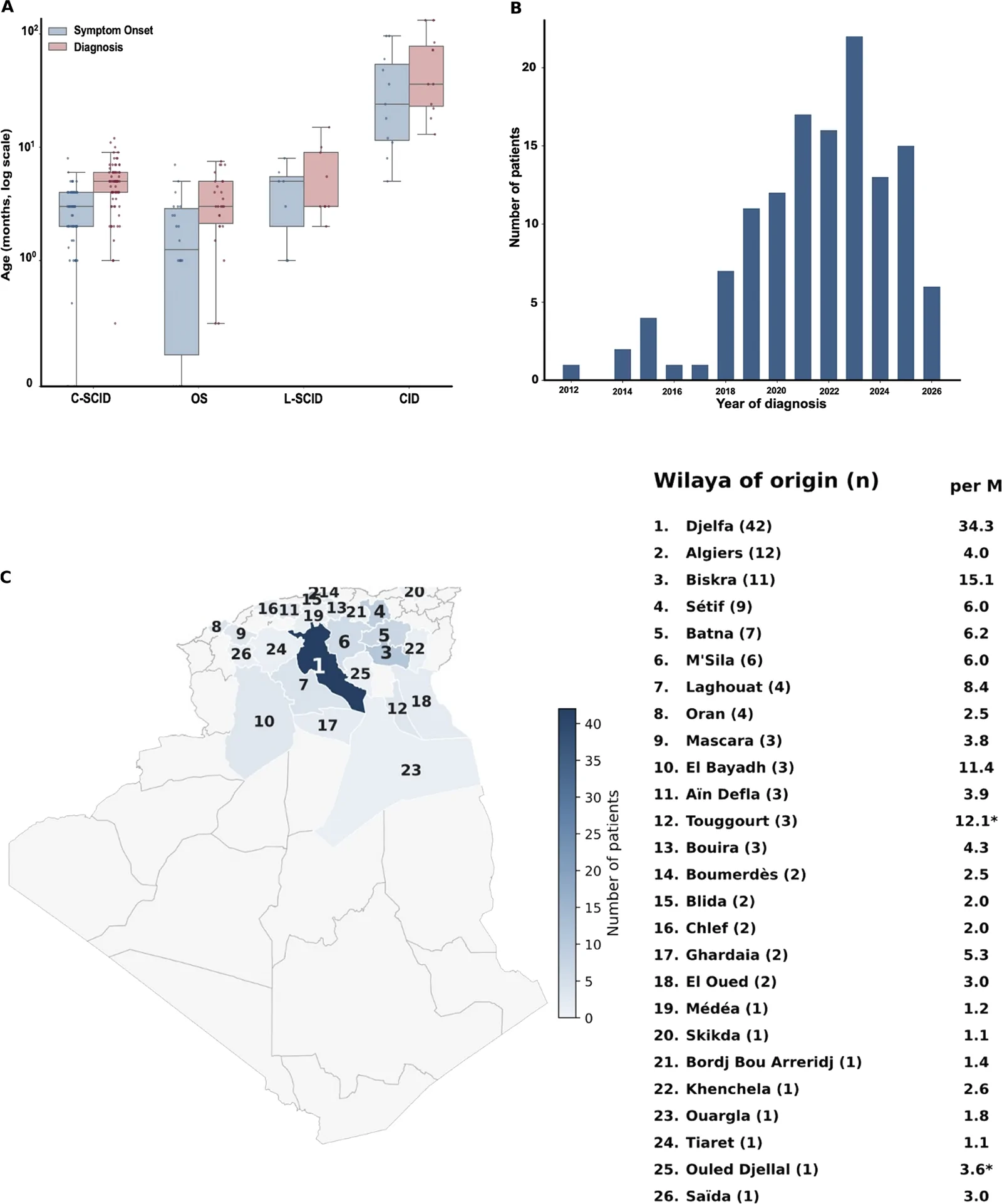
**Demographic and epidemiological characteristics of the study cohort. (A)** Box plots comparing age at onset vs. age at diagnosis across phenotypes. **(B)** Number of new diagnoses per year. **(C)** Geographic distribution of RAG1/RAG2-deficient patients by province (wilaya) of origin.

Median age at symptom onset was 3 mo (interquartile range [IQR], 1.6–4; *n* = 110), diagnostic delay was 2 mo (IQR, 1–4; *n* = 109), and age at diagnosis was 5 mo (IQR, 3–7; *n* = 122) (Fig. S1 A). Annual diagnoses rose markedly from 2018 onward, peaking at 22 in 2023, after a sporadic baseline of 1–4/year (2012–2017) (Fig. S1 B). Patients originated from 27 provinces, concentrated in Djelfa (42/128, 32.8%), followed by Algiers (12, 9.4%), Biskra (11, 8.6%), Setif (9, 7.0%), and Batna (7, 5.5%) (Fig. S1 C).

Patients were classified into four phenotypic categories per PIDTC 2022 definitions (Table 1 and Table S1). c-SCID was most common (*n* = 78, 61%), comprising 55 patients without and 23 with maternal T cell engraftment (c-SCID-MTE); this subdivision appears separately in Table S1 but is combined as a single c-SCID category elsewhere. c-SCID was followed by OS (*n* = 30, 23.4%), CID (*n* = 11, 8.6%), and l-SCID (*n* = 9, 7%).

Phenotype was strongly associated with causal gene (P < 0.001): RAG2 accounted for most c-SCID (68/78, 87.2%), OS was exclusively RAG1 (30/30, 100%), and RAG1 also predominated in l-SCID (8/9, 88.9%) and CID (7/11, 63.6%) (Table S1).

Age at onset, age at diagnosis, and diagnostic delay all differed significantly by phenotype (all P < 0.001; Fig. S1 A): OS had the earliest onset (median, 1.2 mo; IQR, 0.2–2.9), while CID presented later (24.0 mo; IQR, 11.5–54) with a longer diagnostic delay (17 mo; IQR, 12–30) than c-SCID, OS, and l-SCID, which shared a similar 2-mo median delay.

### Spectrum of clinical manifestations

**Figure 1.**
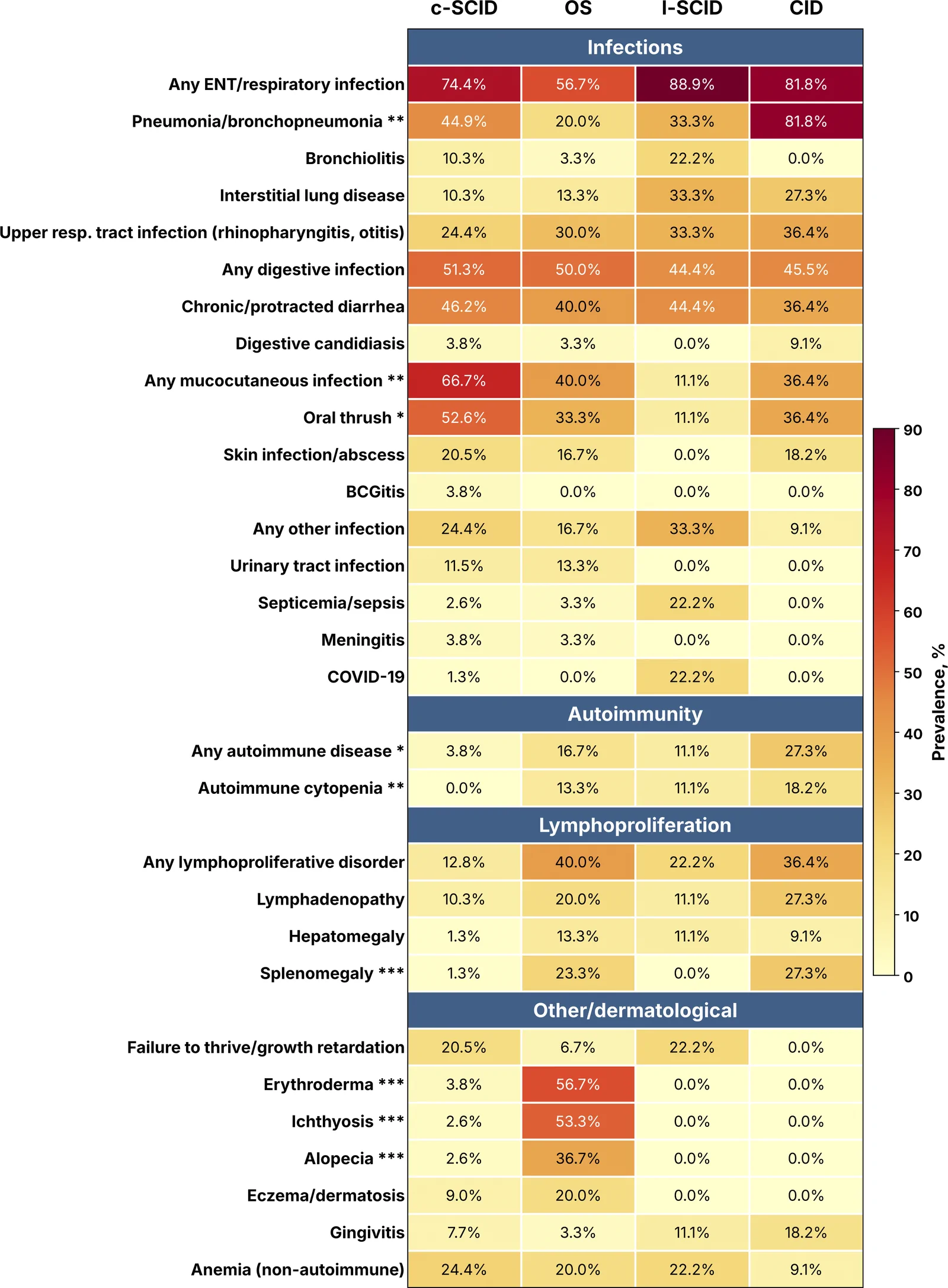
**Prevalence of clinical manifestations by PIDTC 2022 phenotype.** *P < 0.05, **P < 0.01, ***P < 0.001 (chi-squared test across the four phenotype groups). c-SCID, classical SCID; OS, Omenn syndrome; l-SCID, leaky SCID; CID, combined immunodeficiency; ENT, ear nose throat.

Table S2 and Fig. 1 summarize clinical manifestations overall and by PIDTC 2022 phenotype. Sinopulmonary infections were the most common feature (92/128, 71.9%; P = 0.139), especially upper respiratory infections (35/128, 27.3%) and pneumonia (53/128, 41.4%), the latter significantly more frequent in CID (9/11, 81.8% vs. 20–44.9% elsewhere; P = 0.003). Digestive infections affected half the cohort (64/128, 50%), mainly chronic diarrhea (56/128, 43.8%), without phenotype differences (P = 0.969, 0.896). Mucocutaneous infections (69/128, 53.9%) differed significantly by phenotype (P = 0.001), highest in c-SCID (52/78, 66.7%) and lowest in l-SCID (1/9, 11.1%); oral thrush followed a similar pattern (56/128, 43.8%; P = 0.048), while skin infections (23/128, 18%) and BCGitis (3/128, 2.3%) did not differ. Other infections occurred in 28/128 (21.9%; P = 0.475), including urinary tract infections (13/128, 10.2%; P = 0.431) and meningitis (4/128, 3.1%; P = 0.853), neither phenotype dependent. Septicemia (5/128, 3.9%) and COVID-19 (3/128, 2.3%) were both significantly more frequent in l-SCID (22.2% each) than elsewhere (0–3.3% and ≤1.3%, respectively; P = 0.031, P < 0.001).

Autoimmune disorders occurred in 12 patients overall (9.4%), significantly more common in CID (3/11, 27.3%) than c-SCID (3/78, 3.8%; P = 0.031); autoimmune cytopenia specifically was absent in c-SCID but affected up to 18.2% of CID patients (P = 0.007). Lymphoproliferative disorders (28/128, 21.9%) were significantly more frequent in OS (12/30, 40.0%) and CID (4/11, 36.4%) than c-SCID (10/78, 12.8%; P = 0.013). Splenomegaly showed a similarly skewed distribution (11/128, 8.6% overall: 23.3% OS, 27.3% CID, 1.3% c-SCID, 0% l-SCID; P < 0.001), whereas lymphadenopathy (18/128, 14.1%) and hepatomegaly (7/128, 5.5%) did not reach significance (P = 0.326, P = 0.070).

The classic OS triad, erythroderma, ichthyosis, and alopecia, was almost exclusive to this phenotype (56.7%, 53.3%, 36.7%, respectively, vs. ≤ 3.8% elsewhere; P < 0.001). Failure to thrive (20/128, 15.6%), eczema/dermatosis (13/128, 10.2%), gingivitis (10/128, 7.8%), and non-autoimmune anemia (28/128, 21.9%) showed no significant phenotype differences.

### Hematological and immunological characteristics

#### Hematological profile

**Figure S2.**
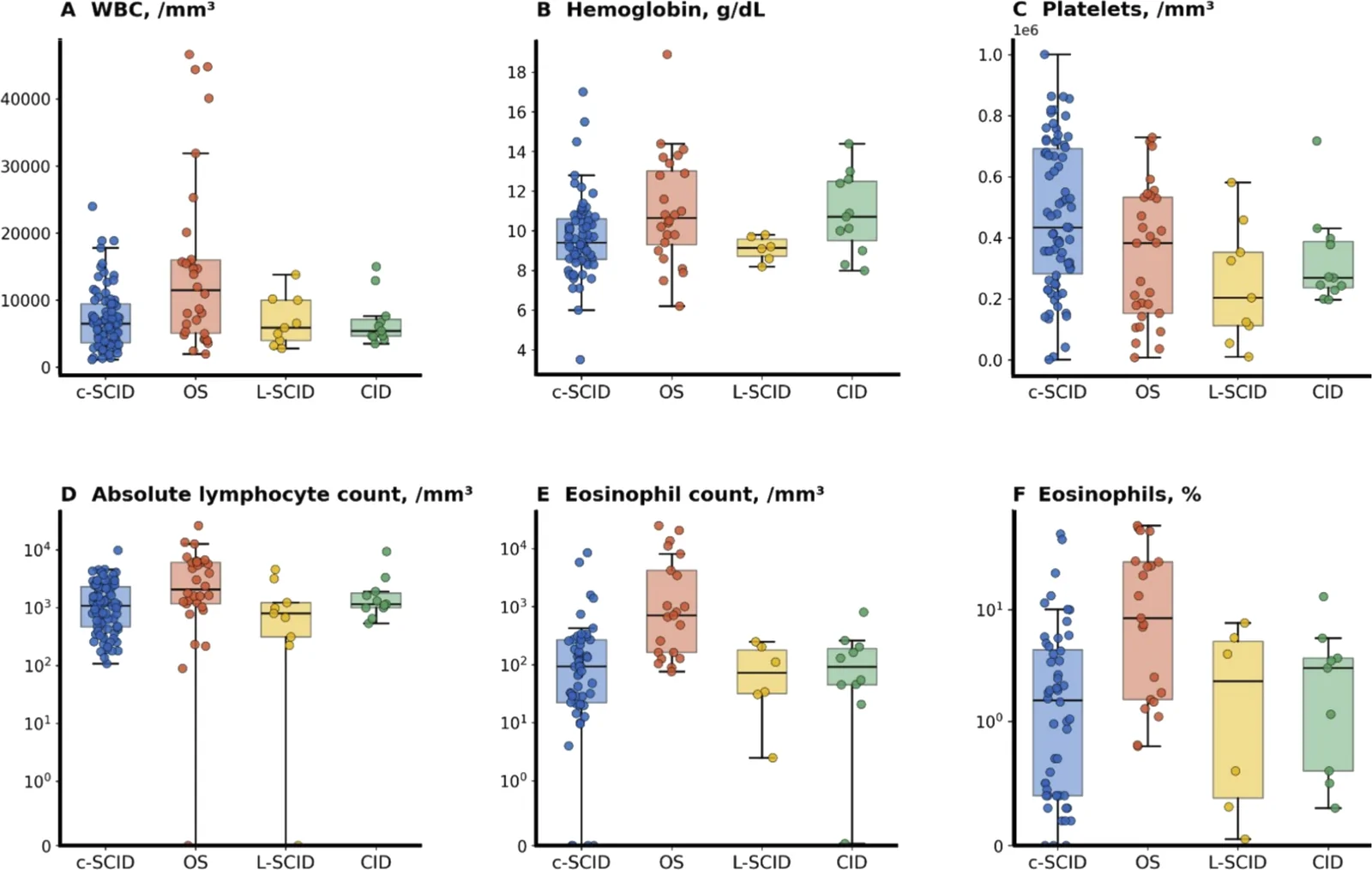
**Hematological characteristics of RAG1/RAG2-deficient patients according to PIDTC 2022 phenotype.** **(A–F)** Dot plots overlaid with boxplots (median and interquartile range) showing (A) white blood cell count, (B) hemoglobin level, (C) platelet count, (D) absolute lymphocyte count, (E) absolute eosinophil count, and (F) eosinophil percentage in patients classified as having classical SCID (c-SCID), Omenn syndrome (OS), leaky SCID (l-SCID), or combined immunodeficiency (CID). Each dot represents an individual patient. White blood cell count, absolute lymphocyte count, absolute eosinophil count, and eosinophil percentage were higher in patients with OS, whereas platelet counts were higher in patients with c-SCID. Hemoglobin levels were lower in patients with c-SCID and l-SCID than in those with OS and CID. Panels D–F use a symmetric logarithmic (symlog) y-axis to accommodate the wide range of values.

Hematological parameters differed significantly by phenotype (Fig. S2 and Table S3). WBC were highest in OS (median 11,470/mm^3^, IQR 5,082–16,000) vs. 5,420–6,495/mm^3^ elsewhere (P = 0.017). Anemia was common overall (82/108, 76%), most frequent in l-SCID (6/6, 100%) and c-SCID (55/67, 82%), and least in OS (14/24, 58%; P = 0.041), mirrored by lower hemoglobin in l-SCID/c-SCID (P = 0.033). Thrombocytopenia occurred in 17/123 (14%), most often in l-SCID (4/9, 44%), never in CID (0/11; P = 0.004), with low platelet counts in l-SCID (204 × 10^3^/mm^3^ vs. 269–434 × 10^3^/mm^3^ elsewhere; P = 0.006). Lymphopenia affected 100/128 (78%), most in c-SCID (68/78, 87%) and least in OS (16/30, 53%; P = 0.002), consistent with higher absolute lymphocyte count (ALC) in OS (2,073 vs. 801–1,142/mm^3^; P = 0.009). Eosinophilia was characteristic of OS (12/21, 57% vs. 0–10% elsewhere; P < 0.001), with higher eosinophil counts (705/mm^3^) and percentages (8.4%) than the rest of the cohort (P < 0.001, P = 0.003).

#### Serum immunoglobulin levels

**Figure 2.**
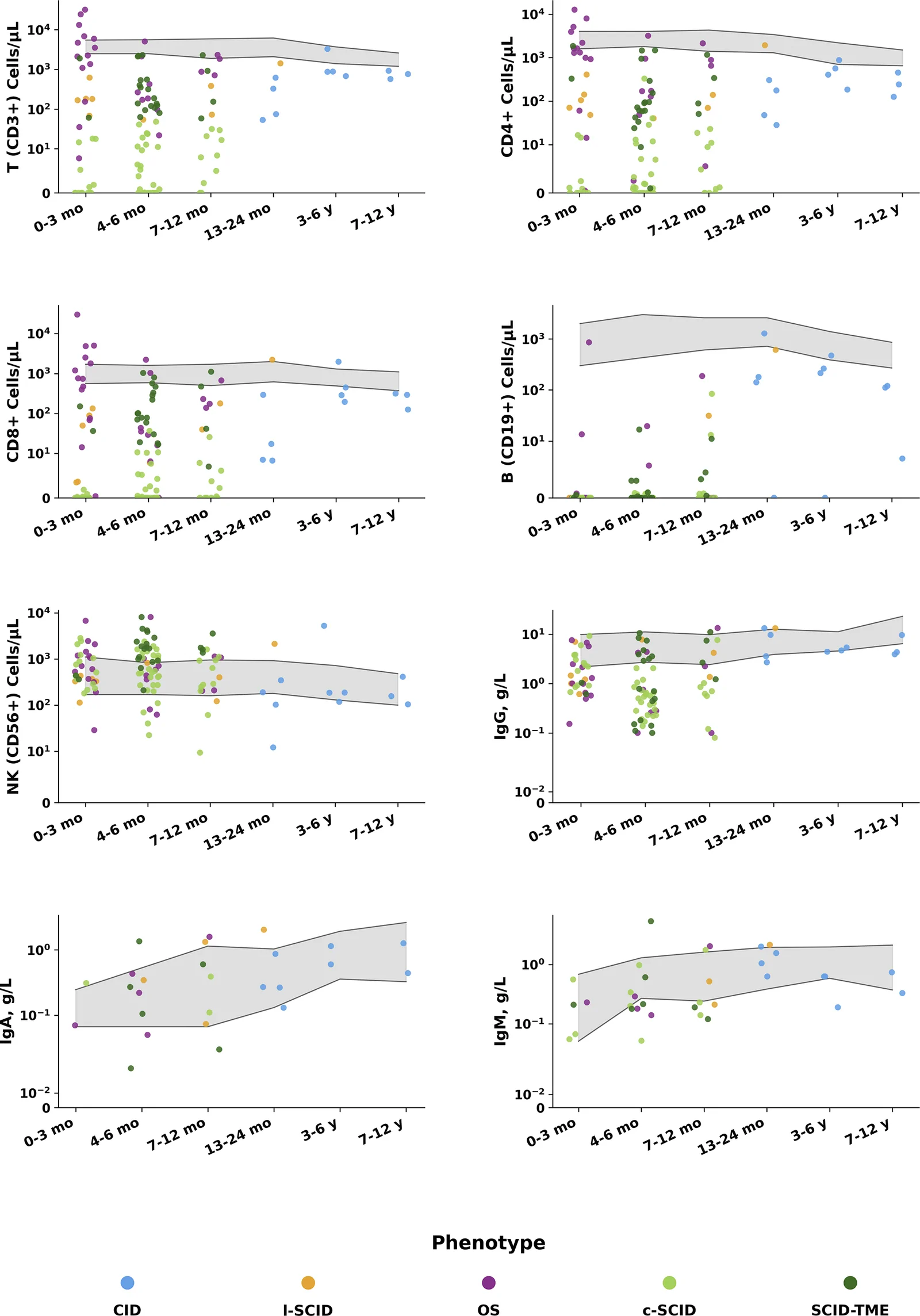
**Absolute lymphocyte subset counts and serum immunoglobulin levels by age at diagnosis and phenotype.** c-SCID, classical SCID; l-SCID, leaky SCID; OS, Omenn syndrome; CID, combined immunodeficiency; y, years. Immunoglobulin levels are assumed to reflect pre-treatment/diagnostic values.

Serum immunoglobulins were profoundly reduced across most of the cohort, consistent with the combined humoral and cellular defect of RAG deficiency, and differed significantly by phenotype (Table S3 and Fig. 2). Median IgG was 1.0 g/L (IQR, 0.4–4.1; *n* = 126) overall, markedly higher in CID (4.5 g/L; IQR, 4.0–8.5) than c-SCID, OS, or l-SCID (0.8–1.4 g/L; P < 0.001). A similar pattern held for IgA (0.4 g/L CID vs. 0.1 g/L elsewhere; P < 0.001) and IgM (0.6 vs. 0.1 g/L; P = 0.001), reflecting relatively preserved humoral immunity in this later-onset phenotype. IgE, measured in only 37 patients, was markedly elevated in l-SCID (median, 34.5 IU/ml; IQR, 17.8–710.3) versus c-SCID (P = 0.002), though data were limited, particularly in CID (*n* = 1).

#### Main lymphocyte subsets

Lymphocyte immunophenotyping showed the expected phenotype-specific T, B, and natural killer (NK) cell patterns (Table S3 and Fig. 2). CD3^+^ and CD4^+^ percentages were near-absent in c-SCID (median 1.5%, 0.6%) vs. higher in OS (62.1%, 33.5%), CID (57.2%, 24.2%), and l-SCID (33.8%, 26.5%; all P < 0.001). CD8^+^ followed a similar pattern, from 0.2% in c-SCID to 22.5% in CID (P < 0.001). CD19^+^ B cells were almost absent (median 0.0%) in c-SCID, OS, and l-SCID but detectable in CID (median 10.4%, IQR 1.8–25.4; P < 0.001), consistent with partial B cell development in this hypomorphic phenotype. CD56^+^ NK cells conversely predominated the residual compartment in c-SCID (97.1%) and l-SCID (62.5%), a smaller fraction in OS (37.8%) and CID (21.3%; P < 0.001).

#### In-depth T and B cell subsets

Within CD4^+^, naive/memory balance differed markedly by phenotype (Table 2): naive-like CD4^+^CD45RA^+^ was low in c-SCID, OS, and l-SCID (0–1.8%) but higher in CID (6.1%; P < 0.001), inversely for CD4^+^CD45RO^+^ (93.7% CID vs. 97.4–100% elsewhere; P < 0.001). Recent thymic emigrant (RTE) CD4^+^ (1.8% vs. 0–0.3%; P = 0.003) and naive CD4^+^ (3.7% vs. 0–0.8%; P < 0.001) were similarly higher in CID, consistent with more preserved thymic output in this later-onset phenotype; central memory T cell (TCM), effector memory T cell (TEM), and terminally differentiated effector memory T cells re-expressing CD45RA (TEMRA) CD4^+^ did not differ (P = 0.427, 0.229, 0.509). Similarly, in CD8^+^, naive-like CD8^+^CD45RA^+^ rose from c-SCID/OS (0.1–0.7%) through l-SCID (18.0%) to CID (42.7%; P < 0.001), with a corresponding CD8^+^CD45RO^+^ decline (P = 0.001); naive CD8^+^ was mainly absent outside CID (0–0.1% vs. 20.3%; P = 0.003). TEMRA CD8^+^ was also higher in CID (13.0% vs. 0.3–3.2%; P = 0.048); TCM/TEM CD8^+^ did not differ (P = 0.343, 0.581).

**Table 2.** Detailed T and B cell subset distribution phenotype

Lymphocyte subset	Overall (*n* = 128)	c-SCID (*n* = 78)	OS (*n* = 30)	l-SCID (*n* = 9)	CID (*n* = 11)	P value
**CD4** ^**+**^ **T cell subsets**						
CD4^+^CD45RA^+^ (naive-like), %	0.3 [0.0–1.8] (*n* = 75)	0.0 [0.0–0.4] (*n* = 30)	0.1 [0.0–0.7] (*n* = 26)	1.8 [0.1–3.4] (*n* = 8)	6.1 [1.8–14.3] (*n* = 11)	<0.001
CD4^+^CD45RO^+^(memory-like), %	99.7 [98.2–100.0] (*n* = 75)	100 [99.6–100.0] (*n* = 30)	99.9 [99.0–100.0] (*n* = 27)	97.4 [95.7–99.5] (*n* = 7)	93.7 [86–98.2] (*n* = 11)	<0.001
RTE CD4^+^, %	0.3 [0.0–1.5] (*n* = 57)	0.3 [0.0–1.1] (*n* = 19)	0.0 [0.0–0.7] (*n* = 21)	0.2 [0.0–1.3] (*n* = 6)	1.8 [1.1–6.2] (*n* = 11)	0.003
Naive CD4^+^, %	0.1 [0.0–1.0] (*n* = 48)	0.0 [0.0–0.1] (*n* = 18)	0.1 [0.0–1.2] (*n* = 15)	0.8 [0.2–20.3] (*n* = 4)	3.7 [0.7–11.4] (*n* = 11)	<0.001
TCM CD4^+^, %	29.2 [7.5–66.7] (*n* = 48)	20.1 [4.7–33.6] (*n* = 18)	37.5 [7.2–68.2] (*n* = 15)	22.1 [6.1–41.3] (*n* = 4)	63.3 [17.7–76] (*n* = 11)	0.427
TEM CD4^+^, %	66.6 [25.6–88.7] (*n* = 47)	79.7 [60.2–89.4] (*n* = 18)	38.0 [25.6–89.2] (*n* = 15)	55.2 [40.4–69.5] (*n* = 4)	36.6 [12.9–80.1] (*n* = 10)	0.229
TEMRA CD4^+^, %	0.3 [0.1–1.6] (*n* = 46)	0.3 [0.0–1.6] (*n* = 16)	0.4 [0.1–2.8] (*n* = 15)	0.3 [0.0–0.6] (*n* = 4)	0.3 [0.1–1.1] (*n* = 11)	0.509
**CD8** ^**+**^ **T cell subsets**						
CD8^+^CD45RA^+^ (naive-like), %	2 [0.0–27.1] (*n* = 76)	0.1 [0.0–9.9] (*n* = 30)	0.7 [0.0–5.4] (*n* = 27)	18 [4.9–29.8] (*n* = 8)	42.7 [26.8–55.9] (*n* = 11)	<0.001
CD8^+^CD45RO^+^ (memory-like), %	97.5 [72.4–100.0] (*n* = 76)	99.5 [83.9–100.0] (*n* = 30)	99 [94.2–100.0] (*n* = 27)	82.2 [70.2–93.6] (*n* = 8)	57.5 [44.2–73.2] (*n* = 11)	0.001
Naive CD8^+^, %	0.1 [0.0–10.8] (*n* = 51)	0.1 [0.0–1.7] (*n* = 18)	0.1 [0.0–0.1] (*n* = 17)	0.0 [0.0–0.6] (*n* = 5)	20.3 [10.8–22.8] (*n* = 11)	0.003
TCM CD8^+^, %	18.6 [1.3–54.6] (*n* = 46)	14.1 [0.1–29.6] (*n* = 17)	34.7 [14.0–82.2] (*n* = 14)	4.0 [1.2–28.6] (*n* = 4)	18.2 [3.5–28.4] (*n* = 11)	0.343
TEM CD8^+^, %	49.1 [14.4–80.5] (*n* = 45)	71.3 [32.8–82.1] (*n* = 16)	27.6 [14.5–75.0] (*n* = 14)	44.2 [7.2–85.4] (*n* = 4)	38.4 [17.8–58.7] (*n* = 11)	0.581
TEMRA CD8^+^, %	3.3 [0.1–14.8] (*n* = 52)	3.2 [0.0–14.9] (*n* = 18)	0.3 [0.0–3.6] (*n* = 18)	1.0 [0.0–13.5] (*n* = 5)	13.0 [8.3–19.0] (*n* = 11)	0.048
**B cell subsets**						
Naive B cells, %	49.8 [42.0–66.6] (*n* = 8)	NA (*n* = 0)	66.5 (*n* = 1)	NA (*n* = 0)	49.8 [45.2–63.6] (*n* = 6)	/
Unswitched memory B cells, %	17.8 [6.9–22.6] (*n* = 11)	NA (*n* = 0)	0.0 [0.0–4.3] (*n* = 3)	NA (*n* = 0)	20.7 [15.1–29.7] (*n* = 7)	/
Switched memory B cells, %	6.0 [3.4–18.0] (*n* = 11)	NA (*n* = 0)	0.0 [0.0–9.3] (*n* = 3)	NA (*n* = 0)	6.0 [4.9–16.0] (*n* = 7)	/
Transitional B cells, %	0.8 [0.3–1.7] (*n* = 11)	NA (*n* = 0)	0.0 [0.0–0.4] (*n* = 3)	NA (*n* = 0)	1.5 [0.7–4.6] (*n* = 7)	/
Plasmablasts, %	1.3 [0.7–3.8] (*n* = 11)	NA (*n* = 0)	0.0 [0.0–0.5] (*n* = 3)	NA (*n* = 0)	1.8 [1.2–3.8] (*n* = 7)	/
Autoreactive B cells, %	11.7 [4.0–24.2] (*n* = 11)	NA (*n* = 0)	0.0 [0.0–1.0] (*n* = 3)	NA (*n* = 0)	15.4 [11.3–37.1] (*n* = 7)	/

Data are presented as median [IQR] (*n*) or, where only one value is available, as that single value. P values (Kruskal–Wallis test) are reported only for T cell subsets, which have adequate sample sizes across all four phenotypes. B cell values are descriptive only and should not be interpreted as a formal statistical comparison across phenotypes. c-SCID, classical SCID; OS, Omenn syndrome; l-SCID, leaky SCID; CID, combined immunodeficiency; RTE, recent thymic emigrants; TCM, central memory T cell; TEM, effector memory T cell; TEMRA, terminally differentiated effector memory T cells re-expressing CD45RA; NA, not available.

B cell subset data were available only with detectable circulating B cells, precluding assessment in c-SCID and severely limiting l-SCID; comparisons are descriptive only (Table 2). In OS (*n* = 1–3), B cells were almost exclusively naive, with negligible memory, transitional, or plasmablast fractions. CID (*n* = 6–7) showed a more diversified compartment: unswitched (20.7%) and switched (6%) memory, transitional (1.5%), plasmablasts (1.8%), and a substantial CD21low fraction (15.4%), consistent with this phenotype’s higher autoimmune burden.

### Mutational spectrum of RAG1/RAG2

#### Overall genotypic landscape of the cohort

**Figure 3.**
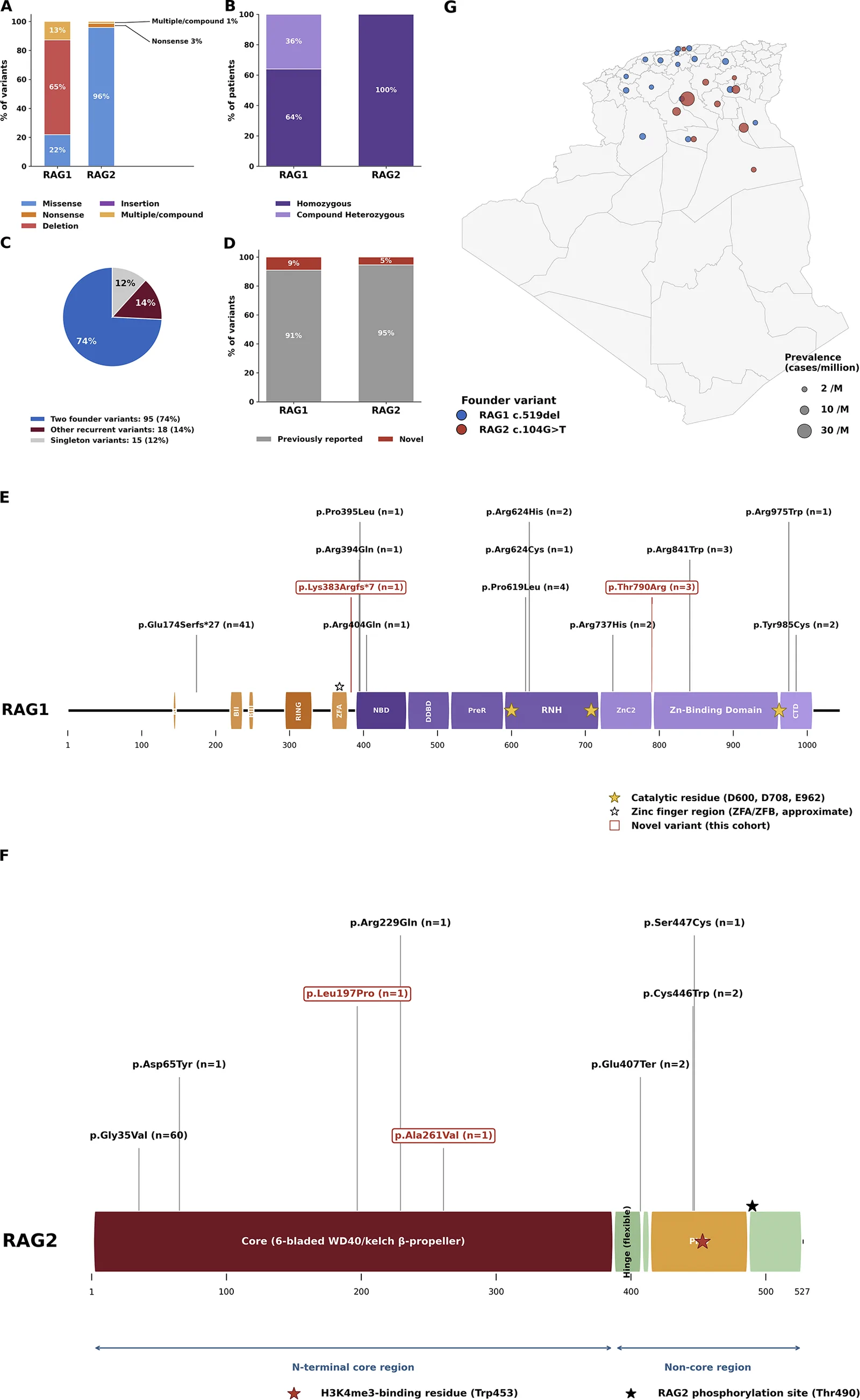
**Molecular spectrum of RAG1/RAG2 variants. (A)** Variant type. **(B)** Inheritance pattern. **(C)** Allelic diversity. **(D)** Variant novelty. **(E and F)** RAG1 and RAG2 protein domain map with cohort variants. **(G)** Geographic distribution of the two founder variants RAG1 c.519del and RAG2 c.104G>T by province. Variant prevalence is expressed as cases per million general population (M) (based on the most recent national census estimates for each province).

Across the 128 patients, 13 distinct RAG1 alleles and 8 distinct RAG2 alleles were identified, corresponding to 24 genotype entries (12 per gene) in 55 RAG1-deficient and 73 RAG2-deficient patients, respectively (Table 3 and Fig. 3). Missense variants were the most frequent variant type, accounting for 18 of 24 genotype entries (83/128 patients, 64.8%), followed by deletion variants (three entries; 37/128, 28.9%; all RAG1), compound deletion/missense genotypes (two entries; 6/128, 4.7%; both RAG1), and a single nonsense variant (2/128, 1.6%; RAG2) (Fig. 3 A). Most genotypes were homozygous (119/128 patients, 93.0%), consistent with the high consanguinity rate in this cohort; the remaining nine patients (7.0%), all with RAG1 variants, were compound heterozygous (Fig. 3 B).

**Table 3.** Complete spectrum of RAG1 and RAG2 variants identified in the cohort, including digenic/oligogenic genotypes

Gene/cDNA change	Protein change	*n* patients	Variant type	Zygosity	ACMG classification	Novelty	Associated phenotype(s)	Recurrent (founder)	Secondary gene(s)
**RAG1 (13 alleles, 12 genotypes)**	​	55	​	​	​	​	​	​	​
c.519del	p.Glu174SerfsTer27	35	Deletion	Homozygous	Pathogenic	Reported	OS (*n* = 26), l-SCID (*n* = 6), C-SCID (*n* = 3)	Yes	/
c.[519del];[1856C>T]	p.[Glu174SerfsTer27];[Pro619Leu]	4	Deletion/missense	Compound heterozygous	Pathogenic/conflicting classifications of pathogenicity	Reported	CID (*n* = 4)	​	/
c.[519del];[1871G>A]	p.[E174Sfs*27];[Arg624His]	2	Deletion/missense	Compound heterozygous	Pathogenic	Reported	OS (*n* = 2)	​	/
c.519del	p.Glu174SerfsTer27	1	Deletion	Homozygous	Pathogenic	Reported	l-SCID (*n* = 1)	Yes	CYBB
c.1146_1147del	p.Lys383ArgfsTer7	1	Deletion	Homozygous	Pathogenic	Novel	c-SCID (*n* = 1)	​	/
c.1181G>A	p.Arg394Gln	1	Missense	Homozygous	Pathogenic/likely pathogenic	Reported	c-SCID (*n* = 1)	​	/
c.[1184C>T];[1211G>A]	p.[Pro395Leu];[Arg404Gln]	1	Missense	Compound heterozygous	Pathogenic/likely pathogenic	Reported	c-SCID (*n* = 1)	​	/
c.1870C>T	p.Arg624Cys	1	Missense	Homozygous	Pathogenic	Reported	c-SCID (*n* = 1)	​	/
c.[2210G>A];[2954A>G]	p.[Arg737His];[Tyr985Cys]	2	Missense	Compound heterozygous	Pathogenic/likely pathogenic	Reported	CID (*n* = 2)	​	/
c.2369C>G	p.Thr790Arg	3	Missense	Homozygous	VUS	Novel	c-SCID (*n* = 3)	​	/
c.2521C>T	p.Arg841Trp	3	Missense	Homozygous	Pathogenic	Reported	OS (*n* = 2), CID (*n* = 1)	​	/
c.2923C>T	p.Arg975Trp	1	Missense	Homozygous	Pathogenic/likely pathogenic	Reported	l-SCID (*n* = 1)	​	/
**RAG2 (8 alleles, 12 genotypes)**	​	**73**	​	​	​	​	​	​	​
c.104G>T	p.Gly35Val	60	Missense	Homozygous	Pathogenic/likely pathogenic	Reported	c-SCID (*n* = 60)	Yes	/
c.104G>T	p.Gly35Val	1	Missense	Homozygous	Pathogenic/likely pathogenic	Reported	c-SCID (*n* = 1)	Yes	PMS2, TLR5
c.104G>T	p.Gly35Val	1	Missense	Homozygous	Pathogenic/likely pathogenic	Reported	c-SCID (*n* = 1)	Yes	ATM
c.193G>T	p.Asp65Tyr	1	Missense	Homozygous	Pathogenic/likely pathogenic	Reported	c-SCID (*n* = 1)	​	/
c.590T>C	p.Leu197Pro	1	Missense	Homozygous	VUS	Novel	CID (*n* = 1)	​	/
c.686G>A	p.Arg229Gln	1	Missense	Homozygous	Pathogenic	Reported	c-SCID (*n* = 1)	​	/
c.782C>T	p.Ala261Val	1	Missense	Homozygous	VUS	Novel	c-SCID (*n* = 1)	​	/
c.1219G>T	p.Glu407Ter	2	Nonsense	Homozygous	Pathogenic/likely pathogenic	Reported	CID (*n* = 2)	​	/
c.1338C>G	p.Cys446Trp	2	Missense	Homozygous	Pathogenic/likely pathogenic	Reported	c-SCID (*n* = 2)	​	/
c.1340C>G	p.Ser447Cys	1	Missense	Homozygous	Pathogenic/likely pathogenic	Reported	l-SCID (*n* = 1)	​	/
c.1340C>G	p.Ser447Cys	1	Missense	Homozygous	Pathogenic/likely pathogenic	Reported	CID (*n* = 1)	​	/
c.1340C>G	p.Ser447Cys	1	Missense	Homozygous	Likely pathogenic	Reported	C-SCID (*n* = 1)	​	IL17RC

Per American College of Medical Genetics and Genomics (ACMG) criteria, most variants were classified as pathogenic or likely pathogenic (119/128 patients, 93.0%); five patients (3.9%) carried a variant of uncertain significance (VUS), and four patients (3.1%) carried a genotype with conflicting evidence of pathogenicity. Two variants, RAG1 c.519del (p.Glu174SerfsTer27) and RAG2 c.104G>T (p.Gly35Val), were markedly overrepresented, together accounting for homozygous form for 98 patients (76.6%) of the cohort (Fig. 3 C); these founding variants are detailed in the following section. Five variants (two in RAG1, three in RAG2), identified in eight patients overall, had not been previously reported in the literature (Fig. 3 D). Four patients, one with a RAG1 genotype and three with a RAG2 genotype, additionally carried a variant in a secondary gene (CYBB, PMS2/TLR5, ATM, or IL17RC), consistent with a digenic or oligogenic genotype; these cases are detailed separately below.

#### Two founder variants

Two variants accounted for most RAG1/RAG2 genotypes: RAG1 c.519del (p.Glu174SerfsTer27) and RAG2 c.104G>T (p.Gly35Val), both previously reported, pathogenic, and classified as candidate founder variants (Table 3), hereafter termed founder variants based on their high recurrence and, for c.104G>T, geographic clustering; this designation is inferred from these patterns rather than confirmed by haplotype/identity-by-descent analysis (see limitations). Homozygous carriage of both accounted for 98/128 patients (76.6%): c.519del in 36/55 RAG1-deficient patients (65.5%) and c.104G>T in 62/73 RAG2-deficient patients (84.9%).

Geographic origin differed markedly between variants (Fig. 3 G). RAG2 c.104G>T carriers were geographically restricted to Djelfa province (39/62, 62.9%; 31.9 cases per million general population, the highest provincial prevalence), with Djelfa and neighboring Biskra (*n* = 7) together accounting for 46/62 (74.2%), and remaining carriers scattered across eight further High Plateau provinces. RAG1 c.519del carriers were considerably more dispersed across 17 provinces spanning the northern coastal and central regions, with no province exceeding 7/36 patients (Algiers, 19.4%; Algiers + Setif, 36.1%). Phenotypic associations also differed: homozygous c.519del was predominantly OS (26/36, 72.2%), with the remainder l-SCID (7/36, 19.4%) or c-SCID (3/36, 8.3%); it was also found in compound heterozygous form with a second RAG1 missense variant in six further patients (four with c.1856C>T, all CID; two with c.1871G>A, both OS). Homozygous c.104G>T, by contrast, was uniformly c-SCID across all 62 carriers.

Two of the 62 homozygous c.104G>T carriers and one of the 36 homozygous c.519del carriers also carried a secondary-gene variant (PMS2/TLR5 and ATM for c.104G>T; CYBB for c.519del), consistent with digenic/oligogenic genotypes, described in Digenic/oligogenic cases. Excluding these three, non-digenic homozygosity for either founder variant accounted for 95/128 patients (74.2%), which, with 18 patients carrying other recurrent variants and 15 with singleton variants, completes the cohort’s allelic diversity landscape.

#### Novel variants

Four variants, two in RAG1 (c.2369C>G, p.Thr790Arg; c.1146_1147del, p.Lys383ArgfsTer7) and two in RAG2 (c.590T>C, p.Leu197Pro; c.782C>T, p.Ala261Val), had not been previously reported in the literature, ClinVar, or Human Gene Mutation Database (HGMD), and were absent from gnomAD; all were identified in six patients overall, homozygous and female (Tables 4 and S4).

**Table 4.** In silico characterization of the four novel RAG1/RAG2 variants

Patients (*n*)	Gene	Genbank accession	HGVS	Variant type	Exon	Protein domain	Zygosity	ACMG classification	gnomAD frequency	ClinVar/HGMD	CADD (phred)	REVEL	SIFT	PolyPhen-2	MutationTaster	Additional predictors
P1, P2, P3 (*n* = 3)	RAG1	NM_000448.3	c.2369C>G (p.Thr790Arg)	Missense	2	Zinc-binding domain ZnC2 (719–791)	Homozygous	VUS	0	Absent	27.5	0.8	D (0.00)	Probably damaging (0.998)	D	Alphamissense: 0.9379; EVE score: 0.664
P4 (*n* = 1)	RAG2	NM_000536.4	c.590T>C (p.Leu197Pro)	Missense	2	Core WD40/kelch-repeat β-propeller domain (1–387)	Homozygous	VUS	0	Absent	28	0.95	D (0.00)	D	D	MutAssessor: M; PROVEAN: D; GERP: 5.7; InterVar: N/A
P7 (*n* = 1)	RAG1	NM_000448.3	c.1146_1147del (p.Lys383ArgfsTer7)	Deletion	2	Junction between the N-terminal non-core region and the beginning of the RAG1 core domain	Homozygous	Pathogenic	0	Absent	N/A	N/A	N/A	N/A	D	MutAssessor: N/A; PROVEAN: N/A; GERP: N/A; InterVar: N/A
P8 (*n* = 1)	RAG2	NM_000536.4	c.782C>T (p.Ala261Val)	Missense	2	Core WD40/kelch-repeat β-propeller domain (1–387)	Homozygous	VUS	0	Absent	25.2	0.722	D (0.00)	Probably damaging (0.997)	D	Grantham: 64 (moderate); PhyloP: 6.41 (highly conserved)

EVE, Evolutionary model of Variant Effect; HGVS, Human Genome Variation Society; VUS, variant of uncertain significance; D, deleterious; N/A, not applicable; P, patient.

**Figure 4.**
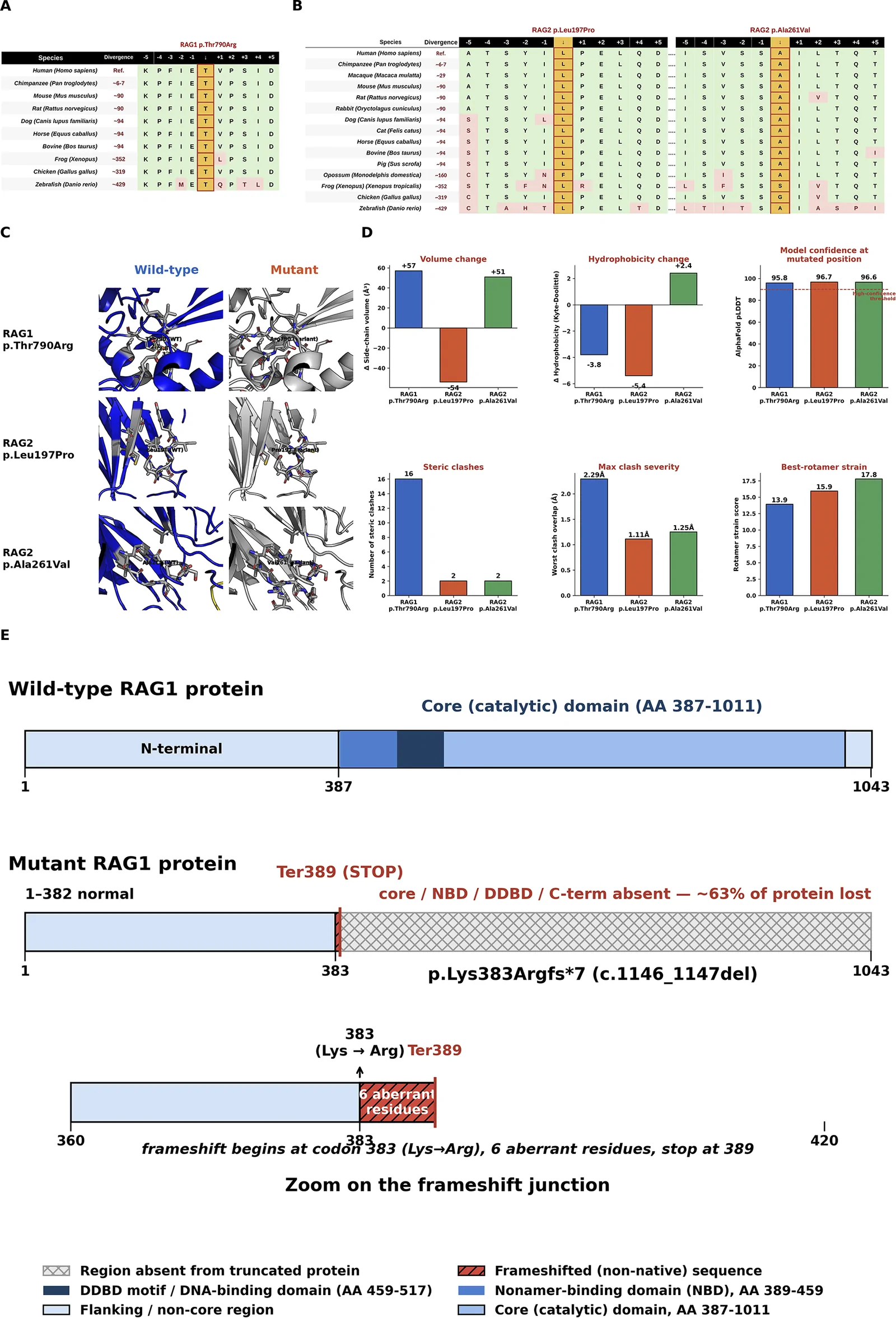
**In silico characterization of RAG1 and RAG2 variants: Evolutionary conservation, structural modeling, and impact on protein architecture. (A)** Interspecies conservation of RAG1 Thr790. **(B)** Interspecies conservation of RAG2 Leu197 and Ala261. **(C)** In silico structural modeling (AlphaFold), wild-type vs. mutant. **(D)** Quantitative biophysical parameters of the missense variants. **(E)** Impact of the RAG1 truncating variant p.Lys383ArgfsTer7 on protein architecture.

In silico and structural analyses (AlphaFold/PyMOL; predicted local distance difference test 95.8–96.7) supported the pathogenicity of the three novel missense variants (Fig. 4). RAG1 p.Thr790Arg (ZnC2 domain; Combined Annotation Dependent Depletion [CADD] 27.5, REVEL 0.80, SIFT deleterious, PolyPhen-2 probably damaging) introduces a bulky, charged arginine into a hydrophobic pocket, causing marked steric clashes. RAG2 p.Leu197Pro and p.Ala261Val (WD40/kelch domain; CADD 25.2/28, REVEL 0.722/0.95, SIFT deleterious, PolyPhen-2 probably damaging) similarly disrupted local structure via proline-induced backbone rigidity and increased side-chain volume, respectively. These single-rotamer models, without energy minimization, are hypothesis-generating; despite convergent evidence, all three remained VUS under ACMG/SCID Variant Curation Expert Panel (SCID-VCEP) criteria (three patients with p.Thr790Arg; one each with p.Leu197Pro and p.Ala261Val).

The remaining variant, RAG1 c.1146_1147del (p.Lys383ArgfsTer7, *n* = 1), was classified as likely pathogenic, introducing a frameshift immediately upstream of the catalytic core, predicted to truncate the entire downstream domain. Clinically, the six carriers (Table S4) presented with c-SCID (*n* = 3, one each with p.Thr790Arg, p.Lys383ArgfsTer7, p.Ala261Val), c-SCID-MTE (*n* = 2, both p.Thr790Arg), or CID (*n* = 1: p.Leu197Pro). Consanguinity (3/6) and family history (3/6) were common. c-SCID patients had profoundly reduced/absent CD3^+^ T cells (0.0–1.7%), versus preserved compartments in CID (49.7–78.2%), consistent with the cohort’s genotype-phenotype gradient.

#### Digenic/oligogenic cases

Four patients (3.1% of the cohort) carried a RAG1 or RAG2 variant together with a variant in a second immune-related gene (PMS2, TLR5, IL17RC, CYBB, or ATM); ACMG classifications ranged from pathogenic/likely pathogenic to VUS (Table S5).

In 3/4 cases, the RAG variant was itself a founder allele, with the phenotype matching the pattern typically associated with that allele: one c-SCID patient carried homozygous RAG2 c.104G>T with homozygous PMS2 c.672G>C (p.Lys224Asn) and TLR5 c.2336G>A (p.Cys779Tyr), both VUS; a second c-SCID patient carried homozygous RAG2 c.104G>T with a compound heterozygous ATM genotype, c.1148C>T (p.Pro383Leu) and c.1235+3_1235+4delAA (p.?), classified likely pathogenic/pathogenic; and an l-SCID patient carried homozygous RAG1 c.519del with a hemizygous CYBB intronic variant, c.1587-22>T, classified likely pathogenic. The remaining case involved the non-founder RAG2 variant c.1340C>G (p.Ser447Cys) with a homozygous IL17RC c.1058A>G (p.Asp353Gly) VUS; this patient presented with c-SCID and died. A fifth patient, also homozygous for RAG2 c.1340C>G, additionally carried a homozygous, synonymous PRKDC c.8784G>A (p.Lys2928Lys) variant classified likely benign; given this classification, we do not attribute this patient’s clinical course to PRKDC.

### Genotype/phenotype correlation

**Figure 5.**
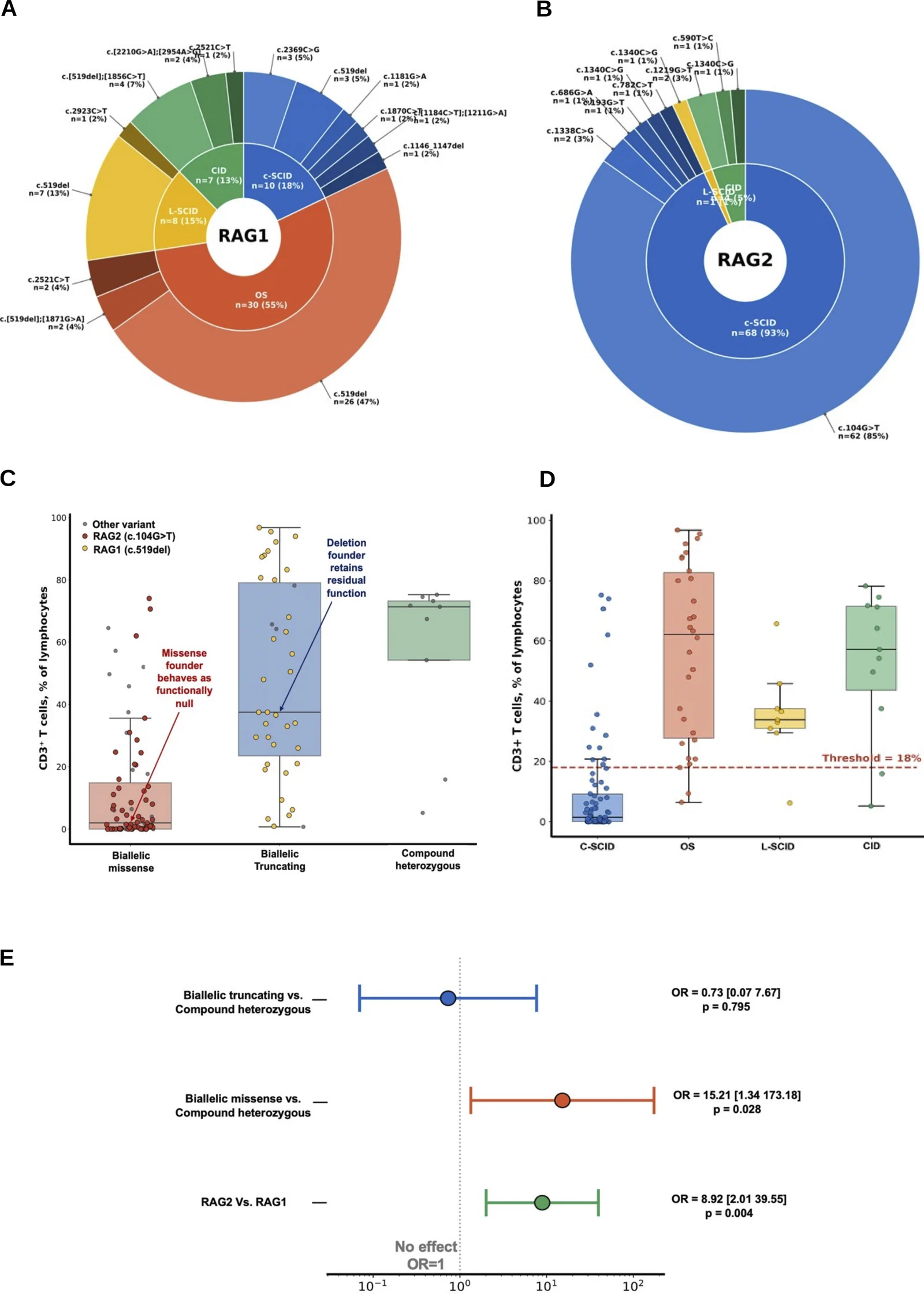
**Genotype–phenotype relationships in RAG1/RAG2 deficiency: From founder mutations to predictive thresholds. (A and B)** Genotype–phenotype architecture of RAG1 and RAG2. **(C)** Residual CD3^+^ T cell output by genotype severity class. **(D)** CD3^+^ as a quantitative discriminator of classical SCID. **(E)** Genotype class and gene as independent predictors.

To explore genotype–phenotype relationships beyond the individual variant, patients were classified into three genotype severity classes by variant type and zygosity: biallelic missense (*n* = 80), biallelic truncating (*n* = 37), and compound heterozygous (*n* = 8) (Fig. 5 and Table S6). Genotype severity class was strongly associated with phenotype (P < 0.001): missense predominated in c-SCID (73/80, 91%), truncating in OS (24/37, 65%) and l-SCID (7/37, 19%), and compound heterozygous in CID (6/8, 75%).

Despite this, genotype did not fully determine phenotype at the individual-variant level (Fig. 5, A and B): among 36 c.519del homozygotes, three phenotypes were observed (OS, 26/36, 72.2%; l-SCID, 7/36, 19.4%; c-SCID, 3/36, 8.3%), whereas c.104G>T was uniformly c-SCID across all 62 homozygotes.

This gradient paralleled T cell findings (Fig. 5 C): median CD3^+^, CD4^+^, and CD8^+^ were lowest with missense (1.8%, 0.9%, 0.4%), intermediate with truncating (37.5%, 28.5%, 14.0%), and highest with compound heterozygous genotypes (71.5%, 35.8%, 11.9%; all P < 0.001), while ALC did not differ (P = 0.324), indicating the relationship reflected T cell proportion rather than overall lymphocyte numbers. The CD3^+^ percentage discriminated c-SCID from other phenotypes with an area under the curve (AUC) of 0.926 and an optimal threshold of 18% (Fig. 5 D).

In a multivariable model (*n* = 127; R^2^, 0.577), genotype severity class and causal gene were independent predictors of c-SCID (Fig. 5 E): relative to compound heterozygous missense, it was associated with higher odds (odds ratio [OR], 15.21; 95% confidence interval [CI], 1.34–173.18; P = 0.028); truncating showed no association (OR, 0.73; 95% CI, 0.07–7.67; P = 0.795); and RAG2 (vs. RAG1) was independently associated with higher odds (OR, 8.92; 95% CI, 2.01–39.55; P = 0.004).

### Kaplan–Meier survival analysis

**Figure 6.**
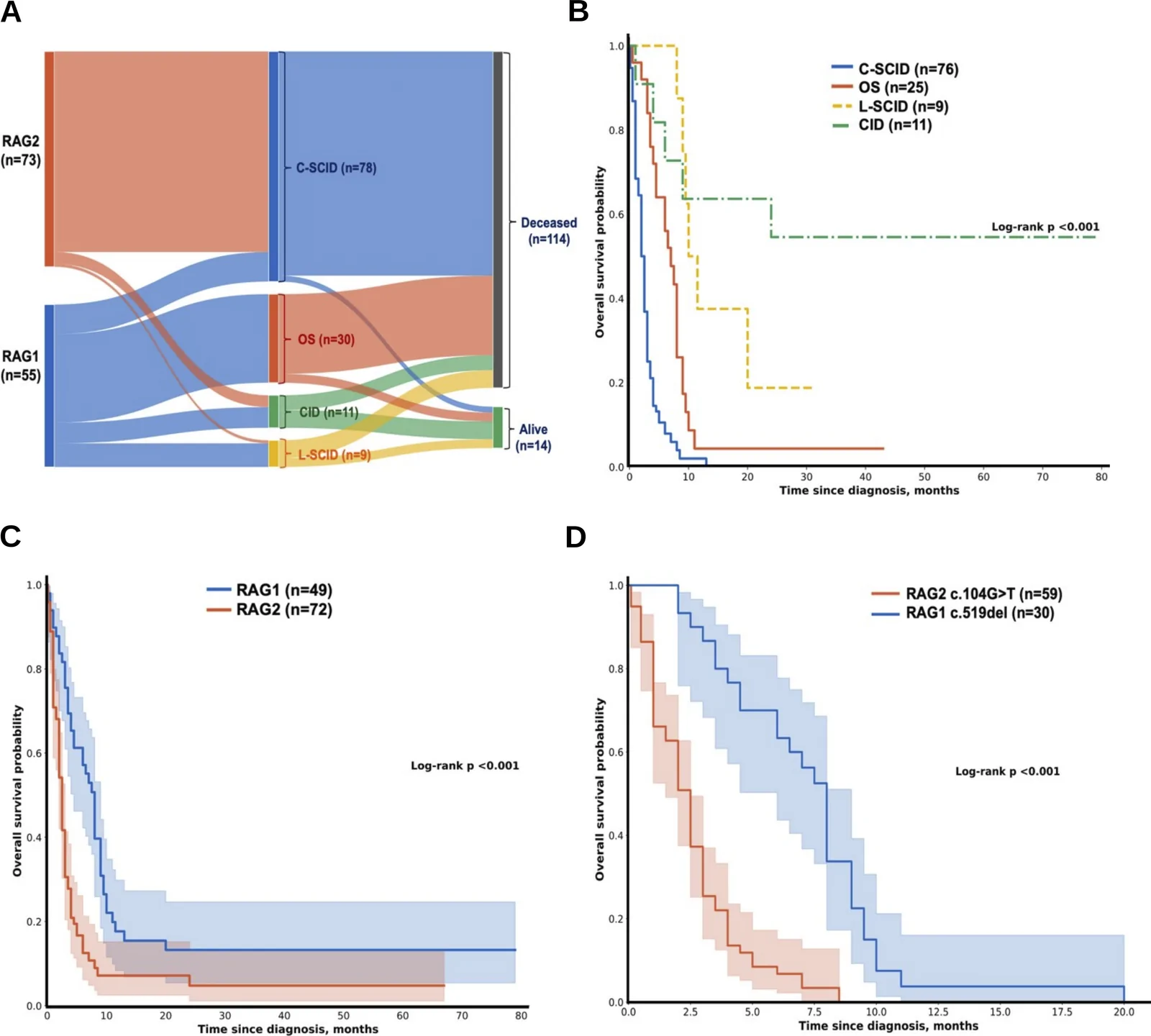
**Overall survival analyses in RAG1/RAG2-deficient patients by phenotype, causal gene, and genotype. (A)** Genotype–phenotype–outcome flow in RAG1/RAG2-deficient patients (*n* = 128). **(B)** Overall survival by phenotype. **(C)** Overall survival by causal gene. **(D)** Overall survival: RAG2 (c.104G>T) vs. RAG1 (c.519del). C-SCID, classical SCID; L-SCID, leaky SCID.

Of 128 patients, 114 had died and 14 remained alive at last follow-up (Fig. 6 A), with deaths and survivorship distributed across both causal genes (RAG1, *n* = 55; RAG2, *n* = 73) and all four phenotypes (c-SCID, *n* = 78; OS, *n* = 30; CID, *n* = 11; l-SCID, *n* = 9). Kaplan–Meier analysis, using age at death for 113/114 deceased patients, showed significant differences by phenotype and gene (log-rank P < 0.001 each; Fig. 6, B and C). Survival declined earliest and most steeply for c-SCID (*n* = 76) and OS (*n* = 25), both reaching very low probability within 2 years, while l-SCID (*n* = 9) and CID (*n* = 11) showed more gradual declines and higher sustained survival. By gene, RAG1-deficient patients (*n* = 49) showed higher survival than RAG2-deficient (*n* = 72) beyond 2 years. A dedicated comparison of the two founder variants likewise showed significant survival differences (log-rank P < 0.001; Fig. 6 D), despite both being associated with high mortality: median survival was markedly shorter for RAG2 c.104G>T (2.5 mo, *n* = 59) than RAG1 c.519del (8.0 mo, *n* = 30), indicating earlier mortality among RAG2 founder carriers.

### Transplantation outcomes

Allogeneic HSCT (all bone marrow transplantation) was performed in 3/128 patients (2.3%): one with CID, from an HLA-genoidentical sibling donor, remained alive at last follow-up; a second with OS, from a haploidentical sibling donor in Turkey, died shortly after; a third with OS underwent HSCT in Spain but was lost to follow-up, precluding outcome assessment.

## Discussion

This study presents the largest reported cohort of RAG1/RAG2-deficient patients from a single North African country (128 cases, 14 years). Two founder variants, RAG1 c.519del and RAG2 c.104G>T, accounted for over three quarters of the cohort in the homozygous state, reflecting high consanguinity (∼70%) and a geographically concentrated founder effect, particularly for RAG2 c.104G>T in Djelfa. Six patients carried four novel variants supported by convergent conservation, in silico, and structural evidence, though functional validation is warranted. Genotype severity class strongly predicted phenotype and T cell output, yet the same homozygous c.519del genotype produced three distinct phenotypes, showing genotype alone does not determine outcome. Despite a short diagnostic delay (median, 2 mo), mortality was extremely high (89.1%), reflecting near-absent access to curative HSCT (3/128). These findings expand the spectrum of RAG deficiency in an underrepresented population and underscore the urgent need for both HSCT access and newborn screening (NBS).

With 128 genetically confirmed patients, this cohort is among the larger reported series of human RAG deficiency worldwide and one of the larger single-country cohorts described to date. Previously published cohorts included 35 patients (25 RAG1, 10 RAG2) from a single Turkish center (10); 31 patients (28 families) from Egypt (16); 26 patients, mostly Palestinian, from an Israeli center (11); 22 Italian patients (12); and 82 patients from 68 families pooled across Slavic countries (13). The recent multiomics characterization by Bosticardo et al. (14), analyzing 157 RAG-deficient patients and 135 controls across multiple international centers, remains larger than this series.

The high consanguinity rate in this cohort (70.3%) sits toward the upper end of MENA-region rates (20–50%), where a North African rate approaching 50% (15, 17), combined with founder mutations, has been proposed to explain the region’s higher CID burden (15). Comparable consanguinity has been reported in other RAG-deficient cohorts: 77% in the predominantly Palestinian/Israeli series (11), with 65% reporting a family history of immunodeficiency, figures closely mirroring this cohort (70.3% and 66.4%, respectively).

The two variants, RAG1 c.519del and RAG2 c.104G>T, accounting for over three quarters of the cohort, represent the first description of founder variants for RAG deficiency in Algeria, fitting an established pattern of recurrent founder alleles in consanguineous populations. Both have previously been reported, but only in isolated cases: c.519del in scattered case reports worldwide (7, 12, 18, 19, 20, 21, 22, 23, 24), and c.104G>T in a handful of patients across several cohorts, including our Egyptian comparator series (7, 16, 25, 26, 27, 28, 29, 30, 31). Their near-exclusive dominance here, rather than mere existence, is the novel finding, consistent with a genuine founder effect rather than a mutational hotspot, echoing a distinct RAG1 frameshift variant, c.256_257del, accounting for 70% of RAG1 patients in Slavic countries, concentrated in Poland’s Vistula watershed (13). MHC class II deficiency, also enriched in North Africa, shows a similar pattern: a 26-bp RFXANK founder deletion described two decades ago was confirmed in 35 additional patients (32), and a distinct RFXANK founder variant occurs in Iran (33); our own Algerian cohort (107 patients, 98 families; unpublished data) was confined to RFXANK c.338-25_338del (77.8%) and RFXAP c.368delG (22.2%). The geographic concentration of c.104G>T around Djelfa, where nearly two thirds of homozygous carriers originated, fits this broader regional pattern across distinct PIDs.

The overall pattern of infectious manifestations was broadly consistent with previously published RAG deficiency series: sinopulmonary infections (71.9%), oral thrush (43.8%), and chronic diarrhea (43.8%) closely paralleled a Turkish cohort of 35 RAG-deficient patients (82.9%, 62.9%, 51.4%, respectively) (10), reinforcing these as near-universal hallmarks across genetically and geographically distinct populations, consistent with infections being the presenting feature in 81.8% of an Italian cohort (12) and in nearly all patients before 2 mo of age in an Israeli/Palestinian series (11). One exception was BCG-related disease, affecting only 3/128 patients (2.3%), all c-SCID. Vaccination status was not systematically recorded, but BCG is part of Algeria’s mandatory national immunization schedule at birth, with 99% coverage, so most patients would be expected to be vaccinated. This contrasts sharply with international data: in a multicenter study of 349 BCG-vaccinated SCID patients from 17 countries, 51% developed BCG-associated complications, over one third disseminated (34). This lower rate may reflect incomplete ascertainment of subclinical/late BCGitis in a high-early-mortality retrospective cohort and limited local mycobacterial culture access. Notably, a Saudi program showed postponing BCG from birth to 6 mo reduced complications in SCID from 46.1% to 2.6% (35), illustrating how vaccination policy alone can produce variation of this magnitude.

Lymphoproliferative manifestations followed a clear phenotype-dependent pattern, affecting 21.9% overall but significantly more frequent in OS (40%) and CID (36.4%) than c-SCID (12.8%; P = 0.013), consistent with lymphadenopathy/hepatosplenomegaly being long recognized as core OS features (10). Splenomegaly showed the sharpest phenotype specificity (23.3% OS, 27.3% CID, vs. 1.3% c-SCID, 0% l-SCID; P < 0.001), a potential bedside discriminator between phenotypes with residual T cell output and the T^−^B^−^NK^+^ SCID profile.

Autoimmune manifestations were markedly less frequent here (9.4% overall) than in previously published series (31.4% [10], 68.2% [12]), dominated by autoimmune cytopenias (7/12, 58.3%) and concentrated in later-onset phenotypes: essentially absent in c-SCID (3.8%) but affecting over a quarter of CID patients (27.3%). This likely reflects an ascertainment gap rather than a true difference: manifestations captured here were limited to overt findings on routine workup, while screening for organ-specific autoimmunity and anti-cytokine autoantibodies was not performed. Broad-spectrum autoantibodies, including neutralizing anti-interferon-α/ω antibodies enriched in CID and l-SCID, are a recognized hallmark of immune dysregulation in RAG deficiency (8, 14) but require dedicated proteomic profiling to detect; the true burden here is therefore likely underestimated.

Differences in T cell reconstitution and survival across PIDTC phenotype groups were expected, since phenotype assignment partly incorporates T cell parameters and the natural history of these categories is already well characterized; we therefore focus interpretation on genotype-level correlates, not circular by construction. Serum immunoglobulins were profoundly reduced across most of the cohort but relatively preserved in CID (median IgG, 4.5 g/L vs. 0.8–1.4 g/L elsewhere), paralleling selective B cell preservation (median, 10.4% vs. near-complete absence elsewhere) and a higher naive-like CD4^+^CD45RA^+^ fraction, a validated thymic-output surrogate in hypomorphic RAG deficiency (36). These findings reflect the graded relationship between residual RAG activity and lymphocyte output, cellular and humoral alike (4, 36): null/near-null activity produces the T^−^B^−^NK^+^ SCID phenotype with no detectable B cell compartment, thymic output, or antibody production, whereas partial activity permits development along both lineages, consistent with hypomorphic RAG variants producing phenotypes extending well beyond c-SCID, including common variable immunodeficiency–like antibody-mediated dysfunction in older children and adults (37, 38).

Beyond the two founder variants, this cohort broadened the molecular spectrum of RAG deficiency, identifying four previously unreported variants and four patients with a co-occurring second-gene variant. The persistence of VUS status for three of the four novel variants, despite convergent conservation, in silico, and structural evidence, illustrates a broader challenge in RAG1/RAG2 variant interpretation. The ClinGen SCID-VCEP, which developed gene-specific ACMG/Association for Molecular Pathology (AMP) criteria for RAG1/RAG2, specifies that only the PS3 criterion (functional evidence) is applicable, requiring a quantitative recombination assay (typically <25% wild-type activity) to upgrade a variant beyond VUS; computational and structural evidence alone cannot substitute (39). All three VUS missense variants fall within SCID-VCEP–defined, PM1-eligible core domains: RAG2 p.Leu197Pro and p.Ala261Val lie within the RAG2 core domain (residues 1–383; WD40/kelch β-propeller), and RAG1 p.Thr790Arg lies within the RAG1 core domain (387–1011; zinc-binding domain, 719–962), each an independently defined PM1-eligible hotspot, providing partial support for all three (39), though a quantitative recombination assay would still be needed to resolve their classification. This underscores functional characterization as a priority for the many RAG missense VUS accumulating in the literature and clinical databases.

The two truncating variants, RAG1 c.1146_1147del (p.Lys383ArgfsTer7) and RAG2 c.1219G>T (p.Glu407Ter), classified likely pathogenic assuming a premature stop abolishes function, warrant a caveat: nonsense-mediated decay (NMD) cannot be assumed, since RAG1/RAG2 are single-exon genes, and the canonical canonical exon junction complex (EJC)-dependent NMD pathway is not expected to degrade their transcripts (40). c.1146_1147del is unreported, while c.1219G>T was previously reported (41). This is not purely theoretical: N-terminal RAG1 frameshifts, predicted null, retain partial recombination activity via downstream internal-methionine reinitiation (23, 42), producing a truncated protein shown to support TCR but not immunoglobulin rearrangement (43). However, this mechanism was characterized for frameshifts far more N-terminal (codons 85, 258) than c.1146_1147del, positioned at the core domain boundary (residues 384–1008) (43), so whether analogous rescue applies is uncertain; regardless, any truncated product would lack most of the catalytic core, so severe impairment remains likely. RAG2 c.1219G>T, previously reported in a Tunisian patient with an atypical agammaglobulinemia-predominant presentation (normal T cell counts, absent B cells) (43), is less clear-cut: located in the hinge region, it preserves the entire WD40/kelch core domain while removing only the C-terminal regulatory region. This is supported by existing functional data: recombination activity for this exact variant was directly assayed in a v-Abl Rag2^−/−^ pro-B cell reconstitution system and retained 2.9% of wild-type activity (44), severely reduced but measurably non-zero, consistent with a hypomorphic rather than null mechanism. Whether this residual activity accounts for the CID phenotype in both carriers here, versus the agammaglobulinemia-predominant presentation previously reported, remains unclear, potentially reflecting modifying genetic or epigenetic factors. Confirmatory RNA-level studies for c.1146_1147del, for which no archival material was available, remain a priority for future work.

The marked phenotypic heterogeneity among patients homozygous for RAG1 c.519del, spanning OS, l-SCID, and c-SCID, reaffirms a principle established over two decades ago: identical RAG1/RAG2 mutations, even within the same kindred, can produce either T^−^B^−^ SCID or OS (7), indicating residual V(D)J activity is necessary but not sufficient to determine presentation. These factors remain poorly understood, but a hypomorphic RAG1 mouse model showed viral pattern-recognition-receptor exposure can precipitate autoantibody production and immune dysregulation on an identical genetic background (8), implicating environmental/infectious triggers alongside stochastic recombination variability and unmeasured genetic modifiers. The independent association of RAG2 with c-SCID in the multivariable model is novel but warrants cautious interpretation given the limited sample size; it is nonetheless biologically plausible, since RAG2 acts as an essential regulatory cofactor for RAG1-mediated recombination (4), so partial RAG2 impairment may affect overall activity more than comparable RAG1 missense changes, though this requires experimental validation. Taken together, genotype constrains but does not determine phenotype in RAG deficiency, and simple, inexpensive flow cytometry (T and B cell percentages) may offer clinically meaningful, real-time information complementing slower genetic confirmation, particularly where diagnosis is often delayed.

The mortality observed in this cohort (89.1% overall, >90% in c-SCID and OS) greatly exceeds that in the RAG deficiency series with modern HSCT access. While survival ≥80% has been achieved in American/European programs (45), only 3/128 patients (2.3%) here underwent HSCT, vs. 73% in Israeli centers (72% survival) (11, 46), 28 predominantly haploidentical Tunisian transplants (65% 5-year survival) (47, *Abstract*), and established Saudi programs (48), reflecting disparities in access rather than disease severity or resources. HSCT is available in Algeria, but eligibility (≥3 years, ≥15 kg, HLA-identical sibling donor only) excludes haploidentical transplantation; given a median diagnostic age of 5 mo and >90% mortality in c-SCID/OS, these criteria effectively preclude timely HSCT for most patients. This cohort illustrates the mismatch: the one patient with an HLA-identical donor was transplanted locally at the Pierre and Marie Curie Center and survives; the patient without a matched sibling, ineligible under Algeria’s genoidentical-only policy, underwent haploidentical transplantation in Turkey and died shortly after.

Given the established survival advantage of HSCT before 3.5 mo (90–94% vs. 66–76% thereafter) (9), restricting eligibility to HLA-identical donors may preclude transplantation during the period of greatest benefit. This cohort’s scale and founder-variant basis support implementing SCID NBS in Algeria, though impact would remain limited without expanded HSCT access, particularly haploidentical transplantation and less restrictive age/weight criteria.

This study has limitations. First, autoimmune manifestations were likely underestimated, as ascertainment relied on retrospective records rather than systematic prospective screening; recruitment from pediatric departments may also have under-represented milder, later-onset (including adult-onset hypomorphic) phenotypes, consistent with, though not conclusive of, the small CID subgroup (*n* = 11). Second, small numbers of l-SCID (*n* = 9) and CID (*n* = 11) patients limited statistical power and widened confidence intervals; the multivariable model, constrained by sparse genotype categories, is hypothesis-generating. The genotype severity classification, based on variant type and zygosity rather than direct measurement of residual recombinase activity, may not fully reflect functional impact, particularly for compound heterozygous missense genotypes. Third, although recurrent RAG1 c.519del and RAG2 c.104G>T strongly suggest founder effects, this was inferred from recurrence and geographic clustering rather than confirmed by haplotype/identity-by-descent analysis. Finally, three of four novel variants remain VUS pending functional recombination assays required by current ClinGen SCID-VCEP criteria; despite conservation, computational, and structural support, their pathogenicity remains unconfirmed.

Despite these limitations, this 128-patient cohort substantially expands the clinical, immunological, and molecular spectrum of RAG deficiency in an underrepresented population. Two founder variants accounted for most cases, supporting targeted first-tier genetic testing, while four novel variants and four digenic/oligogenic cases further broaden the molecular landscape. Genotype severity and a simple CD3^+^ threshold effectively stratified phenotype and prognosis, though phenotypic variability among patients sharing the same founder genotype highlights additional genetic and environmental modifiers. The most important finding is the gap between diagnostic capability and access to curative care: mortality approached 90%, largely reflecting near-absent timely HSCT rather than delayed recognition alone. Improving outcomes will require both NBS and expanded HSCT capacity, as neither alone is likely to substantially reduce mortality.

## Materials and methods

### Study design

This was a retrospective, single-center cohort study conducted at the Laboratory of Medical Immunology, Beni Messous University Hospital Center (CHU Beni Messous), Algiers, Algeria, between 2012 and 2026. This laboratory serves as the principal national reference center for the investigation of IEIs in Algeria, receiving patient referrals from pediatric departments across the entire national territory, which allowed for the ascertainment of a geographically representative, nationwide cohort of RAG1/RAG2-deficient patients despite the single-center design.

Patients were initially evaluated by pediatricians in their referring hospital’s pediatric department whenever a PID was clinically suspected; the referring pediatrician then sent either a peripheral blood sample or the patient directly to this laboratory for sample collection and initial immunological workup, accompanied by a standardized referral form documenting age, sex, province, family history, parental consanguinity, detailed clinical manifestations, and the suspected diagnosis. When findings were consistent with a PIDs, a second blood sample was obtained from the patient, together with samples from both parents after informed consent, to proceed with genetic investigation when available.

### Patients

All patients with a molecularly confirmed, biallelic genotype (pathogenic, likely pathogenic, or variant of uncertain significance VUS) in RAG1 or RAG2, identified through the laboratory’s clinical and genetic database between 2012 and 2026, were retrospectively included, regardless of PIDTC 2022 phenotypic classification (c-SCID, OS, l-SCID, or CID). Genetic confirmation was a mandatory inclusion criterion. As most patients were referred through a pediatric care network, only patients diagnosed in childhood were captured; no adult-onset presentations or patients diagnosed after transition to adult care are represented in this cohort. No further exclusion criteria were applied.

### Phenotypic classification

Demographic, clinical, and follow-up data, including gender, parental consanguinity and its degree, family history of PID, geographic origin (province), age at symptom onset, age at diagnosis, clinical manifestations by organ system, and vital status at last follow-up, were retrospectively extracted from patients’ clinical and laboratory records. Patients were classified into four phenotypic categories: c-SCID, OS, l-SCID, and CID, according to the PIDTC 2022 consensus definitions (6).

### Immunological and hematological evaluation

Complete blood counts with differential leukocyte counts, including ALC and eosinophil count/percentage, were performed on a Convergys X5 automated hematology analyzer (Convergent Technologies). Serum IgG, IgA, and IgM levels were measured by laser nephelometry on BN ProSpec or Atellica systems (Siemens Healthineers), and serum IgE was measured by chemiluminescence immunoassay on an Immulite 2000 XPi analyzer (Siemens Healthineers); immunoglobulin levels were interpreted against age-specific pediatric reference ranges (49). Lymphocyte immunophenotyping was performed by flow cytometry on a BD FACSLyric instrument (BD Biosciences), with data acquired using BD FACSuite software v1.2 and analyzed using FlowJo v10 (BD Biosciences) for extended subset analyses.

Following exclusion of doublets, lymphocytes were identified by CD45 (PerCP-Cy5.5, clone 2D1)/side-scatter gating. T cells were identified as CD3^+^ (FITC, clone SK7) and further subdivided into CD4^+^ (PE-Cy7, clone SK3) and CD8^+^ (APC-Cy7, clone SK1) subsets; within the CD3^−^ population, B cells were identified as CD19^+^ (APC, clone SJ25C1) and NK cells as CD16/CD56^+^ (PE, clones B73.1/NCAM16.2). Lymphocyte subset counts and percentages were interpreted against age-specific pediatric reference ranges (50). Surface expression of HLA-DR (FITC, clone L243) on CD19^+^ B cells (PerCP-Cy5.5, clone SJ25C1) and monocytes was additionally assessed in all patients to exclude MHC class II deficiency as a differential diagnosis.

In a subset of patients, an extended panel further characterized CD4^+^ (APC, clone SK3) and CD8^+^ (PE-Cy7, clone SK1) T cell maturation subsets within CD3^+^ (PerCP-Cy5.5, clone SK7) cells using CD45RA (FITC, clone L48), CD45RO (PE, clone UCHL1), CCR7/CD197 (BV510, clone 2-L1-A), and CD31 (APC-Cy7, clone WM59; BD Pharmingen): naive (CD45RA^+^CCR7^+^), TCM (CD45RA^−^CCR7^+^), TEM (CD45RA^−^CCR7^−^), and TEMRA (CD45RA^+^CCR7^−^) subsets were distinguished by combined CD45RA/CCR7 expression, and RTE were identified within the CD4^+^ compartment as the CD31^+^CD45RA^+^ subset.

B cell maturation subsets were similarly characterized using CD19 (V450, clone SJ25C1), CD27 (PE, clone L128), and IgD (APC, clone IA6-2; BD Pharmingen) to distinguish naive (CD27^−^IgD^+^), non-switched memory (CD27^+^IgD^+^), and switched memory (CD27^+^IgD^−^) B cells; CD24 (PE-Cy7, clone ML5; BD Pharmingen) and CD38 (PerCP-Cy5.5, clone HIT2; BD Pharmingen) to distinguish transitional (CD24^high^, CD38^high^) B cells and plasmablasts (CD24^low/−^, CD38^high^); and CD21 (FITC, clone B-ly4; BD Pharmingen) and CD38 to identify autoreactive B cells, defined as a CD21^low^CD38^low^ subset. Antibodies were from BD Biosciences or BD Pharmingen, as specified above.

T cell proliferative capacity was assessed by a carboxyfluorescein succinimidyl ester (CFSE) dilution assay. Peripheral blood mononuclear cells were labeled with CFSE and stimulated with either phytohemagglutinin or anti-CD3/anti-CD28–coated magnetic beads (Dynabeads, Thermo Fisher Scientific); proliferation was assessed by sequential CFSE dilution within the CD3^+^ T cell population by flow cytometry, with 7-aminoactinomycin D (BD Pharmingen) used to exclude non-viable cells from analysis.

### Molecular and genetic analysis

Genetic diagnosis was established through a combination of approaches. 55 patients underwent whole exome sequencing (WES) in collaboration with the National Institute of Allergy and Infectious Diseases, National Institutes of Health (Bethesda, MD, USA), and 51 patients underwent WES in collaboration with the Department of Paediatrics and Adolescent Medicine, The University of Hong Kong; in both groups, variants identified within RAG1 or RAG2 were subsequently confirmed by Sanger sequencing. For the remaining 22 patients, genetic diagnosis was established locally at our laboratory, either by direct Sanger sequencing of RAG1 and RAG2 (*n* = 15) or by a targeted gene panel covering genes associated with SCID (*n* = 7). Regardless of the sequencing approach, parental samples, obtained as described above, were used for segregation analysis to confirm the biallelic (homozygous or compound heterozygous, in trans) nature of identified variants. Identified variants were classified according to standard ACMG/AMP criteria, incorporating population frequency data (gnomAD), in silico pathogenicity predictors (CADD, REVEL, SIFT, PolyPhen-2, MutationTaster, Alphamissense, MutAssessor, PROVEAN, Grantham,), evolutionary conservation metrics (PhyloP and Genomic Evolutionary Rate Profiling [GERP]), and, for selected novel missense variants, structural modeling using AlphaFold-predicted protein structures with PyMOL-based mutagenesis to assess steric and biophysical consequences of the substitution. Gene-specific ACMG/AMP specifications developed by the ClinGen SCID-VCEP were not applied.

### Statistical analysis

Continuous variables were summarized as medians with IQR and compared across the four PIDTC 2022 phenotypic categories using the Kruskal–Wallis test, given the non-normal distribution of most immunological and hematological parameters. Categorical variables were summarized as counts and percentages and compared across groups using the chi-squared test. The same approach was used to compare three genotype severity classes, defined by variant type and zygosity as biallelic missense, biallelic truncating (nonsense, frameshift, or deletion), or compound heterozygous (one missense and one truncating allele).

A multivariable logistic regression model was used to evaluate genotype severity class and causal gene (RAG1 vs. RAG2) as independent predictors of c-SCID phenotype, with results expressed as odds ratios (OR) and 95% confidence intervals. Receiver operating characteristic curve analysis was performed to assess the discriminative performance of CD3^+^ T cell percentage for c-SCID versus the other three phenotypes combined, with the AUC calculated and the optimal threshold determined by the Youden index.

Overall survival was estimated using the Kaplan-Meier method, with survival time calculated from age at diagnosis to age at death (for deceased patients with date-level data available) or to the most recent follow-up (for patients alive at last contact, censored at that time point). Survival curves were compared across phenotype, causal gene, and founder genotype using the log-rank test.

A two-sided P value <0.05 was considered statistically significant throughout. All statistical analyses were performed using R (RStudio, version 2026.01.0+392; Posit Software, PBC).

### Online supplemental material

This article contains supplementary information, including two supplementary figures (Figs. S1 and S2) and six supplementary tables (Tables S1, S2, S3, S4, S5, and S6). Fig. S1 presents age-at-onset versus age-at-diagnosis distributions by phenotype, the annual number of new diagnoses over the study period, and the national geographic distribution of patients. Fig. S2 presents hematological characteristics by PIDTC 2022 phenotype. Table S1 details the PIDTC 2022 phenotype distribution by causal gene. Table S2 details clinical manifestations by phenotype. Table S3 details hematological and immunological characteristics of the cohort. Table S4 details the clinical and immunological characteristics of the six patients carrying novel RAG1/RAG2 variants. Table S5 details the digenic/oligogenic cases. Table S6 details the genotype severity class in relation to phenotype and functional immune reconstitution.

## Ethics statement

### Ethics approval

This study was approved by the ethics committee of CHU Issad Hassani, Beni Messous, Algiers, Algeria (approval number N°14CE/2026). Written informed consent was obtained from parents or legal guardians of all patients at the time of genetic testing, as part of routine clinical care. The study was conducted in accordance with the Declaration of Helsinki.

## Supplementary Material

Table S1 shows detailed distribution of PIDTC 2022 phenotypes, overall and by causal gene (RAG1 vs. RAG2).

Table S2 shows clinical manifestations of the cohort, overall and by PIDTC 2022 phenotype.

Table S3 shows hematological and immunological characteristics of the cohort.

Table S4 shows detailed clinical and immunological characteristics of the six patients carrying novel RAG1/RAG2 variants.

Table S5 shows digenic/oligogenic cases: patients carrying a RAG1/RAG2 variant plus a variant in a second candidate gene.

Table S6 shows genotype severity class in relation to phenotype and functional immune reconstitution.

## Data Availability

The data underlying this study are not publicly available due to patient privacy issues. The data are available from the corresponding author upon request. All RAG1 and RAG2 variants identified in this cohort have been submitted to ClinVar (https://www.ncbi.nlm.nih.gov/clinvar/; submission IDs SUB16510658, SUB16511119, SUB16511145, SUB16509736).
