# Supplementary material for: The genetic landscape of RAG deficiency: Novel and founder variants in a large Algerian cohort
Source: J Hum Immun. 2026 Oct 26;3(1):e20260168. doi: 10.70962/jhi.20260168 (PMC13701135; doi:10.70962/jhi.20260168)
Supplement: Supplementary file 1 [file jhi_20260168_tables1.docx]

**Table S1. Detailed distribution of PIDTC 2022 phenotypes, overall and by causal gene (RAG1 vs RAG2)**

| **PIDTC 2022 phenotype** | **Overall (n = 128)** | **RAG1 (n = 55)** | **RAG2 (n = 73)** |
| --- | --- | --- | --- |
| c-SCID | 55 (43.0%) | 8 (14.5%) | 47 (64.4%) |
| c-SCID-MTE | 23 (18.0%) | 2 (3.6%) | 21 (28.8%) |
| OS | 30 (23.4%) | 30 (54.5%) | 0 (0.0%) |
| l-SCID | 9 (7.0%) | 8 (14.5%) | 1 (1.4%) |
| CID | 11 (8.6%) | 7 (12.7%) | 4 (5.5%) |

*PIDTC, Primary Immunodeficiency Treatment Consortium. c-SCID, Classical SCID. c-SCID-MTE, classical SCID with Maternal T-cell engraftment. l-SCID, Leaky SCD. OS, Omenn Syndrome. Data are presented as n (%). C-SCID-MTE is reported separately from C-SCID given its distinct immunological presentation.* *Combined, c-SCID and c-SCID-MTE correspond to the single 'c-SCID' category (n=78) used in Table 1 and throughout the main text. Overall chi-squared test comparing the five-phenotype distribution between RAG1 and RAG2:* ***p <0.001****.*
