# Supplementary material for: The genetic landscape of RAG deficiency: Novel and founder variants in a large Algerian cohort
Source: J Hum Immun. 2026 Oct 26;3(1):e20260168. doi: 10.70962/jhi.20260168 (PMC13701135; doi:10.70962/jhi.20260168)
Supplement: Supplementary file 2 [file jhi_20260168_tables2.docx]

**Table S2.** Clinical manifestations of the cohort, overall and by PIDTC 2022 phenotype

| **Manifestation** | **Overall (n=128)** | **C-SCID (n=78)** | **OS (n=30)** | **L-SCID (n=9)** | **CID (n=11)** | **p-value** |
| --- | --- | --- | --- | --- | --- | --- |
| **Sinopulmonary infections** |  |  |  |  |  |  |
| Any ENT/respiratory tract infection | 92 (71.9%) | 58 (74.4%) | 17 (56.7%) | 8 (88.9%) | 9 (81.8%) | 0.139 |
| Pneumonia /bronchopneumonia | 53 (41.4%) | 35 (44.9%) | 6 (20.0%) | 3 (33.3%) | 9 (81.8%) | **0.003** |
| Bronchiolitis | 11 (8.6%) | 8 (10.3%) | 1 (3.3%) | 2 (22.2%) | 0 (0.0%) | 0.213 |
| Interstitial lung disease | 18 (14.1%) | 8 (10.3%) | 4 (13.3%) | 3 (33.3%) | 3 (27.3%) | 0.151 |
| Upper respiratory tract infection (rhinopharyngitis, otitis) | 35 (27.3%) | 19 (24.4%) | 9 (30.0%) | 3 (33.3%) | 4 (36.4%) | 0.784 |
| **Digestive infections** |  |  |  |  |  |  |
| Any digestive infection | 64 (50.0%) | 40 (51.3%) | 15 (50.0%) | 4 (44.4%) | 5 (45.5%) | 0.969 |
| Chronic / protracted diarrhea | 56 (43.8%) | 36 (46.2%) | 12 (40.0%) | 4 (44.4%) | 4 (36.4%) | 0.896 |
| Digestive candidiasis | 5 (3.9%) | 3 (3.8%) | 1 (3.3%) | 0 (0.0%) | 1 (9.1%) | 0.758 |
| **Mucocutaneous infections** |  |  |  |  |  |  |
| Any mucocutaneous infection | 69 (53.9%) | 52 (66.7%) | 12 (40.0%) | 1 (11.1%) | 4 (36.4%) | **0.001** |
| Oral thrush | 56 (43.8%) | 41 (52.6%) | 10 (33.3%) | 1 (11.1%) | 4 (36.4%) | **0.048** |
| Skin infection / abscess | 23 (18.0%) | 16 (20.5%) | 5 (16.7%) | 0 (0.0%) | 2 (18.2%) | 0.503 |
| BCGitis | 3 (2.3%) | 3 (3.8%) | 0 (0.0%) | 0 (0.0%) | 0 (0.0%) | 0.579 |
| **Other infections** |  |  |  |  |  |  |
| Any other infection | 28 (21.9%) | 19 (24.4%) | 5 (16.7%) | 3 (33.3%) | 1 (9.1%) | 0.475 |
| Urinary tract infection | 13 (10.2%) | 9 (11.5%) | 4 (13.3%) | 0 (0.0%) | 0 (0.0%) | 0.431 |
| Septicemia / sepsis | 5 (3.9%) | 2 (2.6%) | 1 (3.3%) | 2 (22.2%) | 0 (0.0%) | **0.031** |
| Meningitis | 4 (3.1%) | 3 (3.8%) | 1 (3.3%) | 0 (0.0%) | 0 (0.0%) | 0.853 |
| COVID-19 | 3 (2.3%) | 1 (1.3%) | 0 (0.0%) | 2 (22.2%) | 0 (0.0%) | **<0.001** |
| **Autoimmune disease** |  |  |  |  |  |  |
| Any autoimmune disease | 12 (9.4%) | 3 (3.8%) | 5 (16.7%) | 1 (11.1%) | 3 (27.3%) | **0.031** |
| Autoimmune cytopenia (thrombocytopenia, hemolytic anemia) | 7 (5.5%) | 0 (0.0%) | 4 (13.3%) | 1 (11.1%) | 2 (18.2%) | **0.007** |
| **Lymphoproliferative disorders** |  |  |  |  |  |  |
| Any lymphoproliferative disorder | 28 (21.9%) | 10 (12.8%) | 12 (40.0%) | 2 (22.2%) | 4 (36.4%) | **0.013** |
| Lymphadenopathy | 18 (14.1%) | 8 (10.3%) | 6 (20.0%) | 1 (11.1%) | 3 (27.3%) | 0.326 |
| Hepatomegaly | 7 (5.5%) | 1 (1.3%) | 4 (13.3%) | 1 (11.1%) | 1 (9.1%) | 0.070 |
| Splenomegaly | 11 (8.6%) | 1 (1.3%) | 7 (23.3%) | 0 (0.0%) | 3 (27.3%) | **<0.001** |
| **Other manifestations** |  |  |  |  |  |  |
| Failure to thrive / growth retardation | 20 (15.6%) | 16 (20.5%) | 2 (6.7%) | 2 (22.2%) | 0 (0.0%) | 0.134 |
| Erythroderma | 20 (15.6%) | 3 (3.8%) | 17 (56.7%) | 0 (0.0%) | 0 (0.0%) | **<0.001** |
| Ichthyosis | 18 (14.1%) | 2 (2.6%) | 16 (53.3%) | 0 (0.0%) | 0 (0.0%) | **<0.001** |
| Alopecia | 13 (10.2%) | 2 (2.6%) | 11 (36.7%) | 0 (0.0%) | 0 (0.0%) | **<0.001** |
| Eczema / dermatosis | 13 (10.2%) | 7 (9.0%) | 6 (20.0%) | 0 (0.0%) | 0 (0.0%) | 0.135 |
| Gingivitis | 10 (7.8%) | 6 (7.7%) | 1 (3.3%) | 1 (11.1%) | 2 (18.2%) | 0.455 |
| Anemia (non-autoimmune) | 28 (21.9%) | 19 (24.4%) | 6 (20.0%) | 2 (22.2%) | 1 (9.1%) | 0.706 |

*Data are presented as n (%). p-values compare the four phenotype groups using Pearson's chi-squared test. Categories are not mutually exclusive; patients may present more than one manifestation within and across categories.* ***c-SCID****, classical SCID;* ***OS****, Omenn syndrome;* ***l-SCID****, leaky SCID;* ***CID****, combined immunodeficiency;* ***PIDTC****, Primary Immunodeficiency Treatment Consortium.*
