# Supplementary material for: The genetic landscape of RAG deficiency: Novel and founder variants in a large Algerian cohort
Source: J Hum Immun. 2026 Oct 26;3(1):e20260168. doi: 10.70962/jhi.20260168 (PMC13701135; doi:10.70962/jhi.20260168)
Supplement: Supplementary file 3 [file jhi_20260168_tables3.docx]

**Table S3. Hematological and immunological characteristics of the cohort.**

| **Variable** | **Overall (n=128)** | **c-SCID (n=78)** | **OS (n=30)** | **L-SCID (n=9)** | **CID (n=11)** | **p-value** |
| --- | --- | --- | --- | --- | --- | --- |
| **Complete blood count** |  |  |  |  |  |  |
| WBC X10^3^/mm3 | 6.7 [4.2-10.9] (n=128) | 6.5 [3.7-9.5] (n=78) | 11.4 [5.0-16.0] (n=30) | 5.9 [4.0-10.0] (n=9) | 5.4 [4.6-7.2] (n=11) | 0.017 |
| Hemoglobin, g/dL | 9.8 [8.6-10.8] (n=108) | 9.4 [8.6-10.6] (n=67) | 10.7 [9.3-13.0] (n=24) | 9.1 [8.7-9.6] (n=6) | 10.7 [9.5-12.5] (n=11) | 0.033 |
| Anemia (Hb <11 g/dL), n/N (%) | 82/108 (76%) | 55/67 (82%) | 14/24 (58%) | 6/6 (100%) | 7/11 (64%) | 0.041 |
| Platelets X10^3^ /mm3 | 384 [217 - 598] (n=123) | 434[282 - 692] (n=74) | 383 [153 - 533] (n=29) | 204 [112 - 352] (n=9) | 269 [236 - 388] (n=11) | 0.006 |
| Thrombocytopenia (<150,000/mm3), n/N (%) | 17/123 (13.8%) | 6/74 (8.1%) | 7/29 (24.1%) | 4/9 (44.4%) | 0/11 (0.0%) | 0.004 |
| ALC, /mm3 | 1,221 [574-2,880] (n=128) | 1,083 [461-2,310] (n=78) | 2,073 [1,172-6,045] (n=30) | 801 [310-1,228] (n=9) | 1,142 [992-1,776] (n=11) | 0.009 |
| Lymphopenia (<3,000/mm3), n/N (%) | 100/128 (78.1%) | 68/78 (87.1%) | 16/30 (53.3%) | 7/9 (77.7%) | 9/11 (81.8%) | 0.002 |
| Eosinophil count, /mm3 | 130 [32-310] (n=89) | 94 [22-270] (n=52) | 705 [162-4,259] (n=21) | 73 [32-180] (n=6) | 92 [45-192] (n=10) | <0.001 |
| Eosinophils, % | 1.9 [0.6-7.1] (n=88) | 1.6 [0.4-4.4] (n=52) | 8.4 [1.6-26.9] (n=21) | 2.3 [0.4-5.2] (n=6) | 3.0 [0.6-3.7] (n=9) | 0.003 |
| Eosinophilia (>500/mm3), n/N (%) | 18/89 (20.2%) | 5/52 (9.6%) | 12/21 (57.1%) | 0/6 (0.0%) | 1/10 (10.0%) | <0.001 |
| **Serum immunoglobulin levels** |  |  |  |  |  |  |
| IgG, g/L | 1.0 [0.4-4.1] (n=126) | 0.8 [0.3-2.5] (n=78) | 1.3 [0.3-5.4] (n=29) | 1.4 [1.2-7.0] (n=9) | 4.5 [4.0-8.5] (n=10) | <0.001 |
| IgA, g/L | 0.1 [0.1-0.1] (n=126) | 0.1 [0.1-0.1] (n=78) | 0.1 [0.1-0.1] (n=29) | 0.1 [0.1-0.3] (n=9) | 0.4 [0.3-0.8] (n=10) | <0.001 |
| IgM, g/L | 0.1 [0.0-0.2] (n=126) | 0.1 [0.0-0.2] (n=78) | 0.1 [0.0-0.2] (n=29) | 0.1 [0.0-0.2] (n=9) | 0.6 [0.4-1.0] (n=10) | 0.001 |
| IgE, IU/mL | 1.0 [1.0-34.5] (n=37) | 1.0 [0.1-1.0] (n=13) | 5.5 [1.0-233.9] (n=20) | 34.5 [17.8-710.3] (n=3) | 0.2 [0.2-0.2] (n=1) | 0.002 |
| **Lymphocyte immunophenotyping** |  |  |  |  |  |  |
| CD3+ T cells, % | 11.6 [0.8-48.4] (n=128) | 1.5 [0.0-9.1] (n=78) | 62.1 [27.7-82.7] (n=30) | 33.8 [30.9-37.5] (n=9) | 57.2 [43.6-71.5] (n=11) | <0.001 |
| CD4+ T cells, % | 3.9 [0.2-29] (n=128) | 0.6 [0.0-4.0] (n=78) | 33.5 [9.0-53.2] (n=30) | 26.5 [16.6-35.3] (n=9) | 24.2 [10.9-32.2] (n=11) | <0.001 |
| CD8+ T cells, % | 2.0 [0.0-14.5] (n=128) | 0.2 [0-3.5] (n=78) | 14.8 [3.8-21.8] (n=30) | 14.1 [2.0-20.1] (n=9) | 22.5 [7.1-27.2] (n=11) | <0.001 |
| CD19+ B cells, % | 0.0 [0.0-0.1] (n=128) | 0.0 (n=78) | 0.0 (n=30) | 0.0 (n=9) | 10.4 [1.8-25.4] (n=11) | <0.001 |
| CD56+ NK cells, % | 82.8 [44.8-99.1] (n=128) | 97.1 [87.2-100] (n=78) | 37.8 [12.7-72.4] (n=30) | 62.5 [47.8-67] (n=9) | 21.3 [10.7-33.1] (n=11) | <0.001 |
| CD4/CD8 ratio | 1.8 [0.9-4.1] (n=97) | 2.0 [0.6-4.5] (n=48) | 1.8 [1.3-4.1] (n=29) | 2.1 [0.9-5.1] (n=9) | 1.4 [0.9-2.8] (n=11) | 0.697 |

*Data are presented as median [interquartile range] (number of patients with available data) for continuous variables. c-SCID, classical SCID; OS, Omenn syndrome; L-SCID, leaky SCID; CID, combined immunodeficiency. ALC, Absolute Lymphocyte Count; p-values compare the four phenotype groups using the Kruskal-Wallis test (continuous variables) or Pearson's chi-squared test.*
