# Supplementary material for: The genetic landscape of RAG deficiency: Novel and founder variants in a large Algerian cohort
Source: J Hum Immun. 2026 Oct 26;3(1):e20260168. doi: 10.70962/jhi.20260168 (PMC13701135; doi:10.70962/jhi.20260168)
Supplement: Supplementary file 4 [file jhi_20260168_tables4.docx]

**Table S4.** Detailed clinical and immunological characteristics of the 6 patients carrying novel RAG1/RAG2 variants

| **Variable** | **P1** | **P2** | **P3** | **P4** | **P5** | **P6** |
| --- | --- | --- | --- | --- | --- | --- |
| **Patient identification & history** |  |  |  |  |  |  |
| Gene / cDNA / protein change | RAG1 c.2369C>G p.Thr790Arg | RAG1 c.2369C>G p.Thr790Arg | RAG1 c.2369C>G p.Thr790Arg | RAG2 c.590T>C p.Leu197Pro | RAG1 c.1146_1147del p.Lys383ArgfsTer7 | RAG2 c.782C>T p.Ala261Val |
| Sex | F | F | F | F | F | F |
| Consanguinity | 2nd degree | 3rd degree | No | 2nd degree | No | No |
| Family history | Yes | No | No | No | Yes | Yes |
| Province | Algiers | Msila | Msila | Bouira | Mascara | El Oued |
| Age at onset, months | 0.7 | 2.0 | 1.3 | 12.0 | 1.0 | 8.0 |
| Age at diagnosis, months | 1.5 | 6.0 | 5.5 | 13.0 | 2.0 | 11.0 |
| PIDTC 2022 phenotype | Classical SCID | Classical SCID with maternal T-cell engraftment | Classical SCID with maternal T-cell engraftment | CID | Classical SCID | Classical SCID |
| **Clinical manifestations** |  |  |  |  |  |  |
| Manifestations (summary) | oral thrush, fungal skin infections, cough | >7 episodes of Bronchopneumonia, rhinopharyngitis,; gastero-enteritis,; buttock abscess post-vaccination, oral thrush,; failure to thrive | diarrhea,; oral thrush; generalized erythroderma, alopecia, anemia | Pharyngitis , bronchopneumonia; autoimmune hemolytic anemia; splenomegaly, hepatomegaly | chronic diarrhea; Eczema, anemia | Staphylococcus ethmoiditis; Measles; anemia |
| **Complete blood count** |  |  |  |  |  |  |
| WBC, /mm3 | 18900 | 7700 | 6700 | 3520 | 7700 | 1320 |
| Hemoglobin, g/dL | 8.9 | 8.0 | 9.4 | 8.0 | 7.6 | 9,7 |
| Platelets, x10^3^/mm3 | 390 | 758 | 277 | 398 | 775 | 467 |
| ALC/mm3 | 945 | 1987 | 2519 | 1637 | 2500 | 136 |
| Eosinophils, % | ND | ND | 2.0 | 3.7 |  | 2.4 |
| **Serum immunoglobulin levels** |  |  |  |  |  |  |
| IgG, g/L | 3.83 | 4.72 | 3.90 | 9.64 | 0.85 | 0.70 |
| IgA, g/L | <0.07 | <0.02 | 0.03 | 0.13 | <0.07 | 0.39 |
| IgM, g/L | <0.04 | <0.11 | <0.10 | 1.05 | <0.05 | 0.23 |
| IgE, IU/mL | ND | <0.1 | <1.0 | ND | ND | ND |
| **Main lymphocyte subsets** |  |  |  |  |  |  |
| CD3+ T cells, % | 0.0 | 4.0 | 52.0 | 49.7 | 0.7 | 1.7 |
| CD3+ T cells, /mm3 | 0 | 79 | 1,310 | 813 | 18 | 2 |
| CD4+ T cells, % | 0.0 | 2.0 | 33.0 | 26.7 | 0.7 | 1.6 |
| CD4+ T cells, /mm3 | 0 | 40 | 831 | 437 | 17 | 2 |
| CD8+ T cells, % | 0.0 | 1.0 | 18.0 | 1.3 | 0.0 | 0.1 |
| CD8+ T cells, /mm3 | 0 | 20.0 | 453 | 21 | 0 | 0 |
| CD19+ B cells, % | 0.0 | 0.1 | 0.0 | 21.5 | 0.0 | 0.2 |
| CD19+ B cells, /mm3 | 0 | 2.0 | 0 | 352 | 0 | 0 |
| CD56+ NK cells, % | 100.0 | 95.9 | 48.0 | 28.8 | 99.2 | 96.1 |
| CD56+ NK cells, /mm3 | 945 | 1,905 | 1,209 | 471 | 2,480 | 131 |
| CD4/CD8 ratio |  | 2.00 | 1.83 | 20.54 | 67.00 | 16.10 |
| **Extended T-cell immunophenotyping** |  |  |  |  |  |  |
| CD4+CD45RA+, % | ND | 0.0 | 0.5 | 1.8 | 2.9 | 0.0 |
| CD4+CD45RO+, % | ND | 100.0 | 99.0 | 98.2 | 95.2 | 100.0 |
| RTE CD4+, % | ND | 0.0 | 0.3 | 0.6 | 1.0 | / |
| Naive CD4+, % | ND | ND | 0.1 | 0.6 | 0.0 | / |
| TCM CD4+, % | ND | ND | 9.4 | 63.3 | 27.2 | ND |
| TEM CD4+, % | ND | ND | 89.5 | 35.8 | 68.5 | ND |
| TEMRA CD4+, % | ND | ND | 1.0 | 0.3 | 4.4 | ND |
| CD8+CD45RA+, % | ND | 0.0 | 2.0 | 17.7 | 0.0 | 23.1 |
| CD8+CD45RO+, % | ND | 100.0 | 98.0 | 81.8 | 69.2 | 76.9 |
| Naive CD8+, % | ND | 0.0 | 0.0 | 12.1 | 15.4 | ND |
| TCM CD8+, % | ND | ND | 0.1 | 25.3 | 0.0 | ND |
| TEM CD8+, % | ND | ND | 99.0 | 38.4 | 69.2 | ND |
| TEMRA CD8+, % | ND | 0.0 | 0.9 | 24.2 | 15.4 | ND |
| **Extended B-cell immunophenotyping** |  |  |  |  |  |  |
| Naive B cells, % | ND | ND | ND | 46.0 | ND | ND |
| Unswitched memory B cells, % | ND | ND | ND | 34.9 | ND | ND |
| Switched memory B cells, % | ND | ND | ND | 14.6 | ND | ND |
| Transitional B cells, % | ND | ND | ND | 0.9 | ND | ND |
| Plasmablasts, % | ND | ND | ND | 1.3 | ND | ND |
| Autoreactive B cells, % | ND | ND | ND | 9.2 | ND | ND |
| Lymphocyte proliferation assay | ND | ND | ND | ND | ND | ND |
| After PHA stimulation for 4 days | ND | ND | ND | 12% (P4) vs 85% (HD) | ND | ND |
| After CD3/CD28 stimulation for 6 days | ND | ND | ND | 10% (P4) vs 82% (HD) | ND | ND |

ALC, Absolute lymphocyte count; HD, healthy donor; D, not done; RTE, recent thymic emigrants; TEM, effector memory T cells; TCM, central memory T cells; TEMRA, terminally differentiated effector memory T cells re-expressing CD45RA.WBC, White blood cells
