# Supplementary material for: The genetic landscape of RAG deficiency: Novel and founder variants in a large Algerian cohort
Source: J Hum Immun. 2026 Oct 26;3(1):e20260168. doi: 10.70962/jhi.20260168 (PMC13701135; doi:10.70962/jhi.20260168)
Supplement: Supplementary file 5 [file jhi_20260168_tables5.docx]

**Table S5. Digenic/oligogenic cases: patients carrying a RAG1/RAG2 variant plus a variant in a second candidate gene**

| **Genes involved** | **cDNA and protein change** | **Zygosity** | **ACMG classification (per gene)** | **Phenotype** | **Consanguinity** | **Vital status** |
| --- | --- | --- | --- | --- | --- | --- |
| RAG2  PMS2  TLR5 | c.104G>T (p.Gly35Val)  c.672G>C (p.Lys224Asn)  c.2336G>A(p.Cys779Tyr) | Homozygous  Homozygous  Homozygous | Pathogenic/Likely pathogenic  Variant of Uncertain Significance  Variant of Uncertain Significance | c-SCID | No | Deceased |
| RAG2  IL17RC | c.1340C>G (p.Ser447Cys)  c.1058A>G (p.Asp353Gly) | Homozygous  Homozygous | Likely Pathogenic,  Variant of Uncertain Significance | c-SCID | Yes | Deceased |
| RAG1  CYBB | c.519del (p.Glu174SerfsTer27)  c.1587-22->T (p?) | Homozygous  Hemizygous | Pathogenic  Likely pathogenic | l-SCID | Yes | Deceased |
| RAG2  ATM | c.104G>T  c.1148C>T(p.Pro383Leu) ; c.1235+3_1235+4delAA (p ?) | Homozygous  Compound heterozygous | Pathogenic  Likely pathogenic/Pathogenic | c-SCID | Yes | Deceased |
