# Supplementary material for: The genetic landscape of RAG deficiency: Novel and founder variants in a large Algerian cohort
Source: J Hum Immun. 2026 Oct 26;3(1):e20260168. doi: 10.70962/jhi.20260168 (PMC13701135; doi:10.70962/jhi.20260168)
Supplement: Supplementary file 6 [file jhi_20260168_tables6.docx]

**Table S6.** Genotype severity class in relation to phenotype and functional immune reconstitution

| **Variable** | **Overall (n=125)** | **Biallelic missense (n=80)** | **Biallelic truncating (n=37)** | **Compound heterozygous (n=8)** | **p-value** |
| --- | --- | --- | --- | --- | --- |
| **SCID phenotype** |  |  |  |  | **<0.001** |
| Classical SCID | 78 (62.5%) | 73 (91.2%) | 4 (10.8%) | 1 (12.5%) |  |
| Omenn syndrome | 28 (22.4%) | 2 (2.5%) | 24 (64.8%) | 2 (25.0%) |  |
| Leaky SCID | 9 (7.2%) | 2 (2.5%) | 7 (18.9%) | 0 (0.0%) |  |
| CID | 10 (8.0%) | 3 (3.75%) | 2 (5.4%) | 5 (62.5%) |  |
| CD3+ T cells, % | 9.4 [0.7-48.0] (n=125) | 1.8 [0.0-14.3] (n=80) | 37.5 [21.0-78.2] (n=37) | 71.5 [64.1-73.5] (n=8) | **<0.001** |
| CD4+ T cells, % | 3.8 [0.1-26.7] (n=125) | 0.9 [0.0-5.5] (n=80) | 28.5 [9.0-43.5] (n=37) | 35.8 [27.6-50.9] (n=8) | **<0.001** |
| CD8+ T cells, % | 2.0 [0.0-14.5] (n=125) | 0.4 [0.0-5.1] (n=80) | 14.0 [3.0-20.0] (n=37) | 11.9 [8.1-22.4] (n=8) | **<0.001** |
| ALC /mm3 | 1214 [582-2868] (n=125) | 1168 [485-2357] (n=80) | 1568 [773-4792] (n=37) | 1053 [902-2425] (n=8) | 0.324 |
| **Vital status at last follow-up** |  |  |  |  | **<0.001** |
| Deceased | 112 (89.6%) | 73 (91.3%) | 35 (94.6%) | 4 (50.0%) |  |
| Alive | 13 (10.4%) | 7 (8,7%) | 2 (5.4%) | 4 (50.0%) |  |

Data are presented as n (%) or median [interquartile range]. p-values compare the three genotype classes using Pearson's chi-squared test (categorical variables) or the Kruskal-Wallis test (continuous variables).
